# Mathematical modeling of active contraction of the human cardiac myocyte: A review

**DOI:** 10.1016/j.heliyon.2023.e20065

**Published:** 2023-09-12

**Authors:** Fisal Asiri, Md Irfanul Haque Siddiqui, Masood Ashraf Ali, Tabish Alam, Dan Dobrotă, Radu Chicea, Robert Daniel Dobrotă

**Affiliations:** a Department of Mathematics, Taibah University, Medina, 42353, Saudi Arabia; b Mechanical Engineering Department, King Saud University, Riyadh, 11421, Saudi Arabia; c Department of Industrial Engineering, College of Engineering, Prince Sattam Bin Abdulaziz University, Al-Kharj, 16273, Saudi Arabia; d CSIR-Central Building Research Institute, Roorkee, 247667, India; e Faculty of Engineering, Lucian Blaga University of Sibiu, 550024, Sibiu, Romania; f Faculty of Medicine, Lucian Blaga University of Sibiu, 550024, Sibiu, Romania; g Carol Davila University of Medicine and Pharmacy Bucharest, Romania

**Keywords:** Mathematical modeling, Mathematical physiology, Active contraction, Heart, Cardiac muscle, Myocyte

## Abstract

**Background and objective:**

In this present research paper, a mathematical model has been developed to study myocyte contraction in the human cardiac muscle, using the Land model. Different parts of the human heart with a focus on the composition of the myocyte cells have been explored numerically to enabling us to determine the interaction of various parameters in the heart muscle. The main objective of the work is to direct the study of the Land model, which has been exploited to simulate the contraction of real human myocytes.

**Methods:**

Mathematical models has been developed based on the Hill model and Huxley model. Myocyte contraction for different scenarios, such as in isometric tension and isotonic tension have been studied.

**Results:**

It is found that increase in stretch, the peak active tension increases, in line with well-established length-dependent tension generation. Five parameters are selected: [Ca^2+^]_T50_, T_ref_, TRPN50, β_0,_ and β_1_, which have been varied in between the range of −50%–100%, to examine the isometric effects of each parameter on the behavior of the tension developed in the intact myocyte cells, with the most sensitive parameter being [Ca^2+^]_T50_.

**Conclusion:**

In conclusion, it is found that the Land model provides a good platform for the analysis of the active contraction of the human cardiac myocyte.

## 1 Introduction

The active contraction of the myocyte is crucial for the pumping function of the heart. The activities involved in the active contraction of the cardiac muscle are complex, hence requiring the development of mathematical models [[1], [2], [3]] [[1], [2], [3]] [3,[3], [3], [4], [5], [6], [7], [8], [9]]. The mathematical models developed for the active contraction of the myocyte provide a unique technique for integrating many pathways to understanding its contraction mechanisms [1,[10], [11], [12], [13], [14], [15], [16], [17]]. In addition, mathematical modeling can also shed light on cardiac function at an organ level [18]. Mathematical and computational modeling have long played a key role in under-standing the physiology of the heart [10,13,16,17,[19], [20], [21], [22], [23]]. Researchers have developed various models of myocyte contraction in the cardiac muscles, such as the Hill model [9] and the Huxley model [24]. Several recent research works have been carried out on human heart muscle function can be found elsewhere [20,[25], [26], [27], [28], [29], [30], [31], [32], [33], [34], [35], [36], [37]].

A recent example is that developed by Land et al. [17], in which they described the development of a cardiac contraction model. The creation of the model was based on original methods for measuring tension generation in human cardiomyocytes. The scientists employed empirical data gathered from cardiac myocytes in humans under normal body temperature conditions. They then integrated their model for myocyte contraction into a comprehensive representation of the entire organ's pumping function. The Land model encompasses complex dynamics like the kinetics of troponin C and tropomyosin, along with a three-state cross-bridge model. This three-state cross-bridge model accurately depicts the changes in the cross-bridge's shape and considers the cell's viscoelastic reaction. Each of these elements is calibrated using empirical data obtained from human cardiomyocytes at body temperature [17]. Additionally, the research enhances the framework for tension generation in the isolated myocyte with exposed fibers. This enhancement aims to replicate the documented tension patterns seen in whole muscles under conditions of isometric tension. Moreover, the objective is to establish a comprehensive model for human tension generation, applicable in simulations spanning various scales. Achieving this entails modifying aspects such as calcium responsiveness, cooperative interactions, and the rates of cross-bridge transitions. This refined model is subsequently employed in simulations that encompass the intricate biventricular operation of the heart. Through this application, the researchers fine-tune the parameterization within the broader context of the entire organ, aiming to attain a normal ejection fraction [5]. This procedure uncovers the distinction in crossbridge cycling rates between myocytes with exposed fibers and those that remain intact. In this work, we will focus on the mathematical modeling of the active contraction of the cardiac myocyte cells. The main objective of this work is to study the Land model in terms of contraction of cardiac myocyte, while also further studying how various parameters of the model, such as the Ca^2+^ profile, Tref, TRPN50, β0 and β1 parameters affect active tension generation in myocyte. The mathematical models studied in this work can also be used to analysis other behaviors of myocyte cells.

## 2 Background of myocyte active contraction

The heart is one of the main organs in the body which facilitates various processes for the effective functioning of the body. It is, however, one of the simplest organs in the human body because its function involves the pumping of blood to different parts of the body through expansions and contractions of its muscles [13]. The contraction and expansion of the human heart are estimated to occur 2.5 billion times in a human lifetime; a mechanical or electrical failure of the heart could lead to either death or slowed biological activities in the body. Electrical signals spread from the sinoatrial node using the bundle of His and Purkinje networks. The Purkinje network has its ends connected to the endocardium on the ventricles. From these connections, an electrical signal can spread using the myocardium [38]. This is the muscle tissue which is the main substance in the heart walls leading to heart contraction. It is, therefore, essential to examine the mechanism of the cardiac muscles through the creation of a mathematical model to allow efficient analysis [6,7,13,19,[39], [40], [41], [42], [43], [44], [45], [46]].

### 2.1 Cardiac muscle cells (myocyte)

The cardiac muscle cells are also known as the cardiac myocytes; they are the muscle cells that make up the cardiac muscle. The cells measure about 100–150 μm long and 15–20 μm in diameter. The muscle cells can translate the electrical signals into mechanical contraction. In this project, the primary focus is on cardiac muscle cells. These cells play an important role in enabling the heart to effectively pump blood and possess two primary functional characteristics [24]. These attributes encompass electrical excitability and mechanical contractility. Following an action potential, voltage-gated Ca^2+^ channels are activated, allowing the entry of Ca^2+^ into the cell. This, in turn, triggers an additional release of Ca^2+^ from the sarcoplasmic reticulum, facilitated by the ryanodine receptors [17]. The increased concentration of Ca^2+^ plays a pivotal role in altering the structure of the myofilaments. This alteration enables the thick filament to bind and connect with the thin filament, consequently initiating muscle contraction. This Ca^2+^ driven alteration of an electrical stimulus into mechanical force is what is known as the excitation-contraction coupling [18].

This muscle cell is composed of myofibrils, which are made up of myofilaments [5,[47], [48], [49]]. The myofibrils further contain repeating microanatomic units referred to as sarcomeres, which represent the core contractile units of the cardiac myocyte. The sarcomere is the region of myofilament structures between two Z-lines, and the distance between Z-lines (i.e., sarcomere length) ranges from about 1.6 to 2.2 μm. The sarcomere is composed of thick and thin filaments [50]. Chemical and physical interactions between the actin and myosin cause the sarcomere to shorten, and therefore the myocyte contracts during the process of excitation-contraction coupling.

### 2.2 Sliding filament theory

The fundamental basis of the sliding filament theory of muscle contraction relies on the interactions taking place between actin and myosin. In terms of their ability to carry action potentials across membrane surfaces, the mechanism of human cardiac muscle cells shares resemblances with nerve cells. However, unlike nerve cells, muscle cells convert these electrical signals into mechanical actions, facilitating the heart's pumping motion. The process of myocyte contraction is intricate, involving the initiation of ionic currents, such as the L-type Ca^2+^ current. This current allows the entry of Ca^2+^ into the cell, leading to the discharge of Ca^2+^ from the intracellular Ca^2+^ reservoir, known as the sarcoplasmic reticulum [24]. Within the structure of the cardiomyocyte, there exist myofibril bundles that contain sarcomeres, which are the functional units responsible for contraction. These sarcomeres comprise thick and thin myofilaments composed of myosin and actin proteins. On a smaller scale, the transfer of calcium between the cytosol and the sarcoplasmic reticulum governs the interaction between these myofilaments. This interaction initiates the shortening of the sarcomeres and drives the process of excitation-contraction throughout the entire cardiac cell [20]. During the excitation phase, the depolarization of the sarcolemma results in the inflow of extracellular calcium into the cardiomyocytes. This elevated intracellular calcium level prompts the sarcoplasmic reticulum to release more Ca^2+^. Subsequently, cytosolic Ca^2+^ ions bind to troponin-C, setting off the activation of the myofilaments [51].

The thin filaments are made up of three proteins which are actin, troponin, and tropomyosin. Each actin has a single myosin binding site. During contraction, myosin heads form a cross-bridge with actin, the main contractile element in a muscle [38]. Contraction is initiated as the cross-bridges connect, generating a force that prompts the movement of the thin filaments alongside the thick filaments. Thee cross-bridges cycling acts as the basis for movement and force generation in the myocyte. Following is a description of the basic cross-bridges muscle contraction cycle.

• The active site on actin is exposed as Ca^2+^ binds to the troponin;
• The myosin heads make cross-bridges with actin;
• The myosin head bends in the event of the power stroke with ADP and phosphate is released;
• A new ATP molecule joins to the myosin head leading to the cross-bridges detaching;
• ATP is hydrolyzed to become the ADP and phosphate. This returns the myosin to a grounded position;

Excitation-contraction coupling in the heart is defined as a process in which an electrical stimulus is changed into a muscle contraction [7,14,16,[51], [52], [53], [54]]. The Ca^2+^ plays a major role in cardiac electrical activity and acts as the activator of myofilaments leading to contraction. The T tubule is depolarized through action potential and leads to the L-type Ca^2+^ opening. This results in the activation of the inward current of Ca^2+^, known as ICa, which flows inward. The entry of Ca^2+^ into the cells triggers the liberation of extra Ca^2+^ via the ryanodine receptors, RyR. This phenomenon is referred to as calcium-induced calcium release (CICR). Subsequently, the disseminated Ca^2+^ binds to the myofilaments. The spread is through the myoplasm which leads to contraction. Finally, the Ca^2+^ exists from the myoplasm and outside the cells through ATP. In order to construct a quantitative model of cross-bridge binding, it is crucial to determine the quantity of actin-binding sites present within an individual cross-bridge. The main assumptions to be made are whether the cross-bridges have a single available site or an endless array of available sites for binding.

### 2.3 Myocyte contraction modeling

Numerous studies have been carried out with the aim of creating a mathematical model that describes the active contraction of cardiac myocytes. This section will provide an overview of the existing body of literature pertaining to the mathematical modeling of human heart myocyte cells during periods of active contraction, as explored in Refs. [7,13,48,53,55]. Numerous mathematical models of human skeletal muscles have been developed. However, none of them have been adopted generally and each of them applies to a specific purpose. Mathematical models form a basis for understanding the functions and interactions in the heart and make it possible to simulate experiments that are not feasible otherwise with current measurement technologies [23,37,42,42,56]. The Hill and Huxley models are two of the core mathematical models for the muscle. Formulated back in 1938, the Hill model has its constraints, particularly in its capacity to deliver a precise depiction of the complete spectrum of muscle parameters' behavior. The Hill model's foundation rests exclusively on the elastic and contractile components, whereas the Hill equation is valuable for constructing and examining diverse muscle models. In contrast, the Huxley model is primarily constructed around cross-bridge kinetics and forms the basis for various models concerning muscle performance. The Huxley model took into account the movement dynamics of muscle filaments and the likelihood of cross-bridge formations. The distribution function, denoted as n(x,t), characterized the distribution of engaged cross-bridges—essentially the rate of interactions between myosin heads and actin filaments—as a function of cross-bridge length, x. The incorporation of force dependency on velocity was inferred through the assumption that the attachment and detachment of cross-bridges are time-dependent procedures. In comparison to the Hill model, the Huxley model offered a more precise examination of active contraction within cardiac muscles. Nevertheless, the precision of the Huxley model is bounded by a range of assumptions introduced during its formulation by Huxley.

Initially, empirical models focused on the mechanics of cardiac muscle involved adaptations of Hill's skeletal muscle models [18]. These adaptations amalgamated a contractile component governed by a hyperbolic force-velocity correlation, a series elastic element for passive response, and a parallel elastic element also contributing passively. The Hunter model was developed in 1999 by Hunter et al. [5] and presented the passive-active mechanics of the cardiac muscle suitable for use in the continuum mechanics models of the entire heart. The model is based on an extensive review of experimental data from a variety of preparations and species, at temperatures from 20 to 27°. Experimental explorations encompassed a diverse array of assessments, spanning isometric tension development, isotonic loading, quick-release, length step, and sinusoidal perturbations. Nevertheless, it is essential to underscore that the model's domain is constrained within the confines of the comparatively limited spectrum of experimental tests that were accessible within that specific timeframe.

The Rice Filament model was developed by Rice et al. [57] and was based on the Hux-ley model of the cardiac muscles. The Rice Filament model approximated the activation and generation of force in cardiac myofilaments using the cytosolic transient of calcium as an input. The fraction of cross-bridges that can bind strongly and produce force depends on the overlap of the thin and thick filament [18]. They defined the single-overlap function for the thick filament SOVF_thic_, as the fraction of thick filament that is opposed to single overlap filament, and this is used to calculate the maximal activated force. On the other hand, the SOVF_thin_ is defined as the single overlap thin filament for interactions of the thin filament length. In this case, the SOVF_thin_ is used in evaluating the binding of calcium to Troponin. Equations (1), (2)) represent the mathematical formulation of the SOVF_thin_ and SOVF_thic_ interactions in the cardiac muscle.

(1) $${SOFV}_{thic}\left(x\right)=\frac{2*{length}_{sovr}\left(x\right)}{{length}_{thick}-{length}_{hbare}}$$

(2) $${SOFV}_{thin}\left(x\right)=\frac{2*{length}_{sovr}\left(x\right)}{{length}_{thin}}$$

where, length_thick_, length_thin,_ and length_hbare_ represent the lengths of the thick, thin filaments and the paler region respectively. Also, x is the sarcomere length, and length_sovr_(x) is the length of the hole single-overlap region [24]. Other factors that feature in the model include passive force, viscosity, a mass term, and a linear series elastic element. Titin contributes to the passive force which functions as a restoring force to reduce the total tension below the rest length. In addition, the viscosity of the muscle filament is set to the experimental mean values, while the mass terms act in reducing the instantaneous changes in the shortening velocity of the muscle [18]. Finally, the elastic factor in the model stimulates the effects of the compliant end connections.

The Land model integrated a dataset obtained from experiments on skinned human cardiac myocytes. This dataset encompassed the passive and viscoelastic traits of isolated myocytes, the relationship between steady-state force and calcium at varying sarcomere lengths, as well as alterations in tension subsequent to rapid length changes or contractions at a constant velocity. To the best of our understanding, this dataset stands as a comprehensive representation of the interplay between length, velocity, and tension generation within human-skinned cardiac myocytes at physiological body temperature [17]. Employing empirical data, the Land model crafted a computational framework for characterizing contraction and passive viscoelastic behavior within human myocytes. This encompassed dynamics related to troponin C and tropomyosin, a three-state cross-bridge model accommodating cross-bridge distortion, and the responsive nature of cellular viscoelasticity [17]. Every constituent is defined through the utilization of authentic empirical data, meticulously gathered from human cardiomyocytes at physiological body temperature. Additionally, they verified that the characteristics of length-related activation at 37 °C resemble those of other species, marked by an adjustment in calcium sensitivity and a rise in maximum tension [17]. The tension generation model within the skinned isolated myocyte was employed to reproduce documented tension patterns observed in whole muscles during isometric contractions. Additionally, it served as a representation of human tension generation for simulations spanning multiple scales. Achieving this entailed adjustments to calcium sensitivity and rates of cross-bridge transitions [17]. The model found application in multi-scale simulations concerning biventricular cardiac performance. It was subsequently fine-tuned in the context of the entire organ, aiming to achieve a healthy ejection fraction as a parameterization goal.

Further, some work described the coupling between mechanical contraction and electrical excitation in cardiac cells, using models that represent excitation-contraction phenomena in single cardiac cells [4,12,14,52,53]. Particular emphasis was given to two recent models of electrophysiology and mechanical contractility, the GPB, and the Rice Filament models, respectively [57]. The Rice Filament model described activation and force generation in cardiac myofilaments and is used to simulate the contraction of the cardiac muscle. On the other hand, the GPB model aimed to simulate excitation-contraction coupling phenomena for the Ca handling and ionic currents in the human ventricular myocyte. Some researchers coupled the GPB, and the Rice Filament models to simulate excitation-contraction coupling phenomena in healthy cardiac cells [3,4,13,56,[58], [59], [60]]. The study further simulated the mechanical contraction of a failing human myocyte.

All these mathematical models possibly have assisted to improve our understanding of the analysis of the active contraction of the human cardiomyocyte [[61], [62], [63], [64], [65], [66], [67], [68]] [[61], [62], [63], [64], [65], [66], [67], [68]] [[61], [62], [63], [64], [65], [66], [67], [68]].

## 3 The general muscle contraction models

In this section, we will briefly review the first two muscle models, the Hill model, and the Huxley model. Both described the time course of single isotonic and isometric con-tractions of the muscles that encompass the dependence of the velocity of load shortening and the relation between force and length [12]. The cardiac muscle is mainly limited by the reproduction of a limited number of contractions of the heart muscle.

### 3.1 The Hill model

The muscle model introduced by A.V. Hill in 1938, known as the Hill model [69], predated the comprehensive understanding of sarcomere anatomy. Hill emphasized that the velocity equation could be utilized to elucidate the pace of shortening and the load encountered during muscle contraction against a consistent load, a phenomenon termed isotonic contraction. This relationship between the contraction of the muscle and constant load is represented in equation (3) below:

(3) $$\left(p+a\right)v=b\left(p_{0}-p\right)\Leftrightarrow v=\frac{b\left(p_{0}-p\right)}{\left(p+a\right)}$$

where *p* is load, *p*_*0*_ is the isometric force, *v* is the velocity of shortening, and *a*, *b* represents the constants obtained by the experimental fitting of data in a force-velocity curve.

In Fig. 1, a standard force-velocity graph for skeletal muscle is depicted. In this case, when velocity is 0, the force or tension, *p*, experienced by the muscle is *p*_*0*_. The value of *p*_*0*_ represents the tension generated by the muscle when the length is constant. The force that is generated by the muscle at a constant length is referred to as the isometric force. The value of *p*_*0*_ is dependent on time in cardiac muscle but constant for skeletal muscle. Therefore, when *p* = *a*, *v* = *bp*_*0*_*/a*, which is the maximum speed at which a muscle can shorten.

**Fig, 1:**
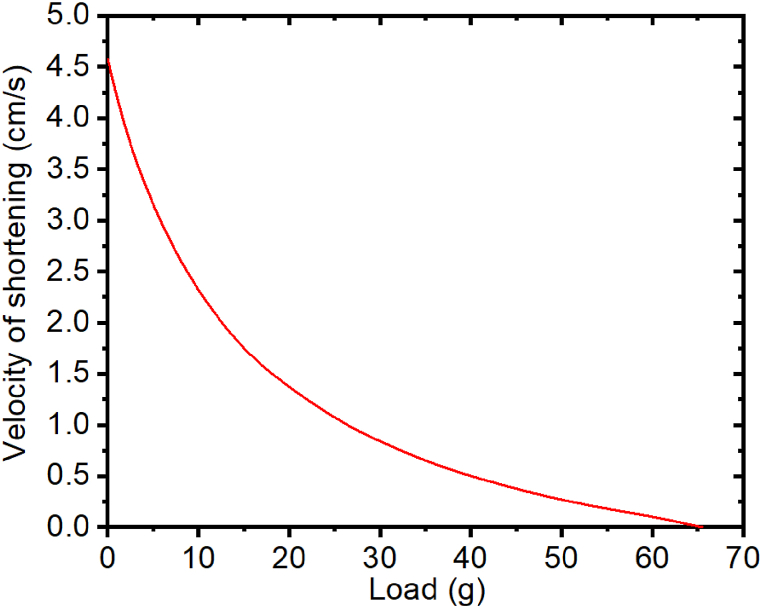
The relationship between the load on a muscle and the velocity of contraction. Parameters used: a = 14.35 g, b = 1.03 cm/s, *p*_0_ = 65.23.

Next, we will describe how Hill derived equation (3). Let *x* be a measure of the muscle shortening with dimensions of length, i.e., cm. Hill observed that when a muscle shortens it emits heat. So, let *ax* be the heat emission from shortening with dimensions of work, i.e., g · cm, where *a* depends on the cross-section of the muscle. If *p* is the load lifted by the muscle, the work done is *px*. Therefore, the total energy (work) is *(p* + *a)x*, and so the rate of energy will be given as (eq. (4))

(4) $$\left(p+a\right)\frac{dx}{dt}=\left(p+a\right)v$$

where *v* = *dx/dt* and is the velocity of shortening. It turns out that *v* is a linear function of *p* (eq. (5)) and it makes sense that we want:

(5) $$v=0⟺p=p_{0}$$

(*p0*: = initial load, i.e., muscle does not move, known as the Tetanus state, given in eq. (6)). We also have:

(6) $$v=v_{\max}⟺p=0\text{,}$$

(Velocity is maximal when, for instance, the arm has no load). So, we have eq. 7

(7) $$\left(p+a\right)v=b\left(p_{0}-p\right)$$

This agrees with the experimental results found by Hill, which were that the rate of energy is a linear function of *p*, increasing as *p* diminishes and becoming zero when *p* = *p*_*0*_ (isometric contraction). We have *(p* + *a)v* = *b(p*_*0*_ *− p)*, where *b* is a constant of the absolute energy liberation. Equation (8), the force-velocity relationship, is a rectangular hyperbola with asymptotes at *p* = *−a* and *v* = *−b*, since

(8) $$v=\frac{b\left(p_{0}-p\right)}{p+a}$$

Now, the Hill model can be tested to determine whether it behaves in a similar way to experimental findings. We model a contractile element in series with a parallel elastic element. The l and x will represent the lengths of the contractile and elastic elements respectively so that the total length of the fiber is *L* = *l + x*. So, it becomes clear that *v* = *− dl/dt*. Assuming that the force generated by the elastic element is a function of its length, *p* = *p(x)*, we can derive a differential equation (eqs. (9), (10), (11), (12), (13))) for the time dependence of *p*:

(9) $$\frac{dp}{dt}=\frac{dp}{dx}\frac{dx}{dt}\text{,}$$

(10) L=l+x

(11) $$\begin{array}{rr} \frac{dx}{dt} & =\frac{dL}{dt}-\frac{dl}{dt}\text{,} \end{array}$$

(12) $$\frac{dp}{dt}=\frac{dp}{dx}\left(\frac{dL}{dt}-\frac{dl}{dt}\right)$$

(13) $$⟺\frac{dp}{dt}=\frac{dp}{dx}\left(\frac{dL}{dt}+v\right)=\frac{dp}{dx}\left(\frac{dL}{dt}+\frac{b\left(p_{0}-p\right)}{p+a}\right)\text{.}$$

Hill assumed that the elastic element is linear, and thus *p* = *α(x − x*_*0*_*)*, where *x*_*0*_ is its resting length. Hence $\frac{dp}{dx}=\alpha$, can be written as in eq. (14);

(14) $$\frac{dp}{dt}=\alpha \left(\frac{dL}{dt}+\frac{b\left(p_{0}-p\right)}{p+a}\right)$$

To solve this, we consider the case where a muscle initially at rest is put into isometric tetanus. Because the tension is measured isometrically, the muscle length is held fixed so $\frac{dL}{dt}=0$. Substituting this into the above equation, we get (eq. (15))

(15) $$\frac{dp}{dt}=\alpha \left(\frac{b\left(p_{0}-p\right)}{p+a}\right)\Rightarrow \frac{p+a}{p_{0}-p}dp=\alpha bdt\text{.}$$

Equation (15), can be solved analytically. We have (eq. (16), (17), (18), (19), (20))):

(16) $$\frac{p+a}{p_{0}-p}dp=\alpha bdt\text{,}$$

(17) $$\Rightarrow \int \frac{p+a}{p_{0}-p}dp=\int \alpha bdt\text{,}$$

(18) $$\Rightarrow \int \left(\frac{p-p_{0}}{p_{0}-p}+\frac{a+p_{0}}{p_{0}-p}\right)dp=\int \alpha bdt\text{,}$$

(19) $$\Rightarrow \int \left(-1+\frac{a+p_{0}}{p_{0}-p}\right)dp=\alpha bt\text{,}$$

(20) $$\Rightarrow -p-\left(a+p_{0}\right)\log\left(p_{0}-p\right)=\alpha bt+C$$

Using the initial condition *p*(0) = 0 we find C as follows (eqs. (21), (23))),

(21) $$C=-\left(a+p_{0}\right)\log\left(p_{0}\right)\text{.}$$

So, the complete solution is

(22) $$-p-\left(a+p_{0}\right)\log\left(p_{0}-p\right)+\left(a+p_{0}\right)\log\left(p_{0}\right)=\alpha bt\text{,}$$

i.e.

(23) $$-p-\left(p_{0}+a\right)\log\left(\frac{p_{0}-p}{p_{0}}\right)=\alpha bt\text{,}$$

which describes the time course of the change in tension.

Next, it is worthwhile to consider a step increase in length where it is shown that the Hill model does not describe well all the aspects of muscle behavior. Assume a muscle is held at its isometric position p0 and then its length suddenly drops. From experimental results, it is expected that the tensions will decrease and then slowly rise back to *p*_0_. We assume that the length of the muscle (eq. (24)) is a function of time,

(24) $$L\left(t\right)=L_{0}+L_{1}-L_{0}H\left(t\right)$$

where *L*_*0*_ is a constant, denoting the magnitude of the length step, *L*_*0*_ + *L*_*1*_ is the length just before the drop in length, and *L*_*1*_ is the length immediately after. *H(t)* is the Heaviside function, defined as (eq. (25))

(25) $$H\left(t\right):=\left\{ \begin{array}{ll} 0, & t<0\\ 1/2, & t=0\\ 1 & ,t>0 \end{array}\right.$$

The Heaviside function, or Unit step function, is a discontinuous function whose value is zero for a negative argument and one for a positive argument. It can be equivalently characterized as (eq. (26));

(26) $$H\left(t\right)=\int_{-\infty }^{t}\delta \left(s\right)ds$$

where *δ(s)* is the Dirac delta function with the half-maximum convention. The Dirac delta function is defined as (eq. (27));

(27) $$\delta \left(x\right):=\left\{ \begin{array}{ll} +\infty , & x=0\\ 0, & x\neq 0 \end{array}\right.$$

together with the additional constraint that it must satisfy $\int_{-\infty }^{+\infty }\delta \left(x\right)dx=1\text{.}$ Furthermore, if we differentiate equation (24) concerning time, we obtain (eq. (28));

(28) $$\frac{dL}{dt}=-L_{0}\delta \left(t\right)$$

and inserting this back into $\frac{dp}{dt}=\alpha \left(\frac{dL}{dt}+\frac{b\left(p_{0}-p\right)}{p+a}\right)$ yields (eq. (29)),

(29) $$\frac{dp}{dt}=\alpha \left(-L_{0}\delta \left(t\right)+\frac{b\left(p_{0}-p\right)}{p+a}\right)$$

with the initial condition *p*($0^{-}$) = *p*_*0*_. Integrating from *t* = *−ε to t* = *ε* and letting *ε* → 0, we have (eq. (30), (31)));

(30) $$\begin{array}{rr} p\left(0^{+}\right)-p\left(0^{-}\right) & =-\alpha L_{0}\text{,} \end{array}$$

(31) $$p\left(0\right)=p_{0}-\alpha L_{0}$$

so, we rewrite the initial value problem (eq. (32)) as follows,

(32) $$\frac{dp}{dt}=\alpha \left(\frac{b\left(p_{0}-p\right)}{p+a}\right)t>0$$

It is readily seen from the definition of *H(t)* and that of *L(t)* can be written as (eq. (33));

(33) $$L\left(t\right)=\left\{ \begin{array}{ll} L_{0}+L_{1}, & t<0\\ \frac{1}{2}L_{0}+L_{1}, & t=0\\ L_{1}, & t>0 \end{array}\right.$$

Using this characterization of *L(t)* as a piecewise function, we can easily solve equation (32), in this case as we did in the previous case; by evaluating the differential equation at each of the sections of the piecewise function. So, we have (eq. (34), (35), (36), (37)));

(34) $$-p-\left(a+p_{0}\right)\log\left(p_{0}-p\right)=\alpha bt+k$$

using the initial condition *p*(0) = *p0 − αL0*,

(35) $$-\left(p_{0}-\alpha L_{0}\right)-\left(a+p_{0}\right)\log\left(p_{0}-p_{0}+\alpha L_{0}\right)=k$$

(36) $$-2p_{0}+\alpha L_{0}-a\;\log\left(\alpha L_{0}\right)=k$$

(37) $$-p-\left(a+p_{0}\right)\log\left(p_{0}-p\right)=\alpha bt-2p_{0}+\alpha L_{0}-a\;\log\left(\alpha L_{0}\right)$$

The implicit solution proposed in this research doesn't correspond with empirical findings concerning tension recovery following a rapid length reduction. An important limitation arises from the presumption of the force-velocity connection, which introduces a marked constraint. In general, the Hill model's effectiveness is hindered by its incapability to accurately represent all dimensions of muscle behavior due to its dependency on elastic and contractile components. Nonetheless, the Hill equation retains its fundamental role in formulating and scrutinizing various alternative muscle models.

### 3.2 A simple cross-bridges model: The Huxley model

This section offers a description of the Huxley model, which is one of the basic models for the contraction of the cardiac muscles. Assuming that the cross-bridges can bind to an actin-binding site at position *x*, where *x* represents the length of the thin filament to the binding site from the cross-bridges, the binding of the cross-bridges to the binding site can occur either at position *x* > 0 or *x* < 0, where they exert a contractile force at *x* > 0 and an expanding force at *x* < 0. In addition, the cross-bridges do not exert any force on the binding site at *x* = 0. The displacement of the cross-bridges at position *x* is equivalent to a value of *x* units. Huxley established the model by assuming that each of the cross-bridges tends to bind with a unique actin-binding site and thus can be linked to a particular value of *x*.

The next assumption made in this model is that the binding of the cross-bridges occurs in a bounded interval −*x*_0_ < *x* < *x*_0_, and ρ is regarded as the number of cross-bridges with displacement x with ρ being a constant independent of the interval. In addition, the variable *n(x, t)* is the fraction of the cross-bridges that are bounded with a displacement *x*. That is, *n(x* = *0, t)* is the fraction of all cross-bridges which are bounded with no generation of force at time *t, n(x* > *0, t)* is the fraction of all those exerting a contractile force, and *n(x* < *0, t)* is the fraction of all those exerting an expanding force. Another assumption here is that there exist two disjoint states U and B, unbounded and strongly bounded respectively, and two constant functions *f(x)* and *g(x)* regulating the rates of detachment and attachment can be written as in equation (38);

(38) $$U\underset{s\left(x\right)}{\overset{f\left(x\right)}{⇄}}B$$

The law of mass action in chemistry tells us that the rate of a chemical reaction is directly proportional to the product of the concentrations of the reactants. From this, and our assumptions in equation (39), it follows that

(39) $$\frac{dn}{dt}=\left(1-n\right)f\left(x\right)-g\left(x\right)n$$

The number of actin-binding sites which are bounded between *a, b* is given by (eq. (40));

(40) $$\rho \int_{a}^{b}n\left(x,t\right)dx\text{.}$$

Moreover, the alteration rate of this quantity is determined by both the interactions of the cross-bridges and the flows across the confines of the range *[a, b]*. At the point *x* = *a*, the outflow of cross-bridges from the region is represented as ρv(t)n(a, t), whereas at *x* = *b*, the inflow of cross-bridges into the region is denoted as *ρv(t)n(b, t)*. In this context, v(t) signifies the actin filament's contraction velocity relative to the myosin filament. For the sake of coherence, it is assumed that *v* > *0* corresponds to muscle contraction (shortening).

To calculate the rate of change, we use the fact that *n* = [*B*] and so 1 − n = [*U*], and equation (39). Hence, we will have relationship as follows (eq. (41), (42), (43), (44)));

(41) $$\rho \frac{d}{dt}\int_{a}^{b}\;n\left(x,t\right)dx=\rho \int_{a}^{b}\;\frac{d}{dt}n\left(x,t\right)dx$$

(42) $$=\rho \int_{a}^{b}\;\frac{\partial n}{\partial t}+\left(v\nabla \right)ndx\text{,}$$

(43) $$=\rho \int_{a}^{b}\;\left(1-n\right)f\left(x\right)-g\left(x\right)ndx+\rho \int_{a}^{b}\;\left(v\left(t\right)\nabla \right)ndx$$

(44) $$=\rho \int_{a}^{b}\;\left(1-n\right)f\left(x\right)-g\left(x\right)ndx+\rho \int_{a}^{b}\;v\left(t\right)\frac{\partial n\left(x,t\right)}{\partial x}dx\text{.}$$

(The first equality is justified because the integral is over *x*, not over *t*). Now the integral can be dropped since *a* and *b* are arbitrary, thus yielding equation (45).

(45) $$\frac{\partial n}{\partial t}-v\left(t\right)\frac{\partial n}{\partial x}=\left(1-n\right)f\left(x\right)-ng\left(x\right)$$

Next, we assume that the sarcomere fiber is moving with constant velocity and that *n* is balanced (eq. (46)), i.e. $\frac{\partial }{\partial t}n\left(x,t\right)=0$, then we get

(46) $$-v\frac{dn}{dx}=\left(1-n\right)f\left(x\right)-ng\left(x\right)\text{.}$$

We can solve this for the cases when (i) *v* is small, and (ii) when *v* is large.

(i) Here we see that quasi-steady-state solution (shown in eq. (47), (48), (49), (50), (51))), $n\left(x\right)=\frac{f}{f+g}$ since v ≈ 0.

(47) 0=(1−n)f(x)−ng(x)

(48) 0=f(x)−nf(x)−ng(x)

(49) 0=f(x)−n(f(x)+g(x)),

(50) $$n=\frac{f\left(x\right)}{f\left(x\right)+g\left(x\right)}$$

(ii) Note that *v* is constant.

(51) $$-v\frac{dn}{dx}=\left(1-n\right)f\left(x\right)-g\left(x\right)n⟺\frac{dn}{dx}-n\frac{f+g}{v}=\frac{-f}{v}$$

we can solve this differential equation by using the technique of an integrating factor. We first find the integrating factor (given eq. (52), (53)));

(52) $$I=e^{\int \rho dx}=e^{\int -\frac{f+g}{v}dx}=e^{-\int \frac{f+g}{v}dx}$$

(53) $$n\left(x\right)=e^{\int \frac{f+g}{v}dx}\int -\frac{f}{g}e^{-\int \frac{f+g}{v}dx}$$

We observe that at low velocities, the force is greatest, while at high velocities the force is less due to the small amplitude of *n(x)*. Next, we suppose that each time one cross-bridge is bound, one ATP loses a phosphate and releases energy as follows (eq. (54));

(54) (1−n(x,t))f(x)=(1−n)f(x)

is the rate of unbounded (U) cross-bridges becoming attached for a given *x* so we integrate to consider all *x* and so *φ* is the total energy released counting each cross-bridge (eq. (55)):

(55) $$\varphi \left(t\right)=\rho \epsilon \int_{-\infty }^{\infty }\left(1-n\right)f\left(x\right)dx\text{.}$$

We do the same with force. Individual force at *x* is *r(x)n(x, t)*, *ϵ* is the energy released by one ATP. So the total force is (eq. (56));

(56) $$p=\rho \int_{-\infty }^{\infty }r\left(x\right)n\left(x,t\right)dx\text{.}$$

Remark, we can also suppose that the cross-bridges is like a linear spring, generating a restoring force, i.e., *r(x)* = *kx, k* constant, and so the total force made by the muscle will be (eq. (57));

(57) $$p=\rho \int_{-\infty }^{\infty }r\left(x\right)n\left(x,t\right)dx\text{.}$$

From this, we observe that at zero velocity the isometric force is (eq. (58));

(58) $$p_{0}=\rho \int_{-\infty }^{\infty }r\left(x\right)\frac{f\left(x\right)}{f\left(x\right)+g\left(x\right)}dx\text{.}$$

Choices for *f(x)* and *g(x*).In order to obtain quantitive models, Huxley chose *f(x)* and *g(x)* to be (eq. (59), (60)));

(59) $$f\left(x\right)=\left\{ \begin{array}{ll} 0, & x<0\\ f_{1}\frac{\pi }{h}, & 0\leq x\leq h\\ 0, & x>0 \end{array}\right.$$

and

(60) $$g\left(x\right)=\left\{ \begin{array}{ll} g_{2}, & x\leq 0\\ g_{1}\frac{x}{h}, & x>0 \end{array}\right.$$

where *f*_*1*_*, g*_*1,*_ and *g*_*2*_ are constants. It makes sense if we consider it as *f(x)* being the rate of attachment at a given *x*. Since the muscle is assumed to be moving, no cross-bridges are attached.

Now, we can easily obtain the steady-state solution of the differential equation for *n(x)*. Let *n*_*1*_*, n*_*2*_*, n*_*3*_ be, the steady-state solutions in the regions *x* ≤ *0, 0* ≤ *x ≤ h* and *h < x* respectively. Then *n*_*1*_ is the solution of the equation. We have (eq. (61)):

(61) $$-v\frac{dn}{dx}=\left(1-n\right)f\left(x\right)-ng\left(x\right)\text{.}$$

**Fig. 2:**
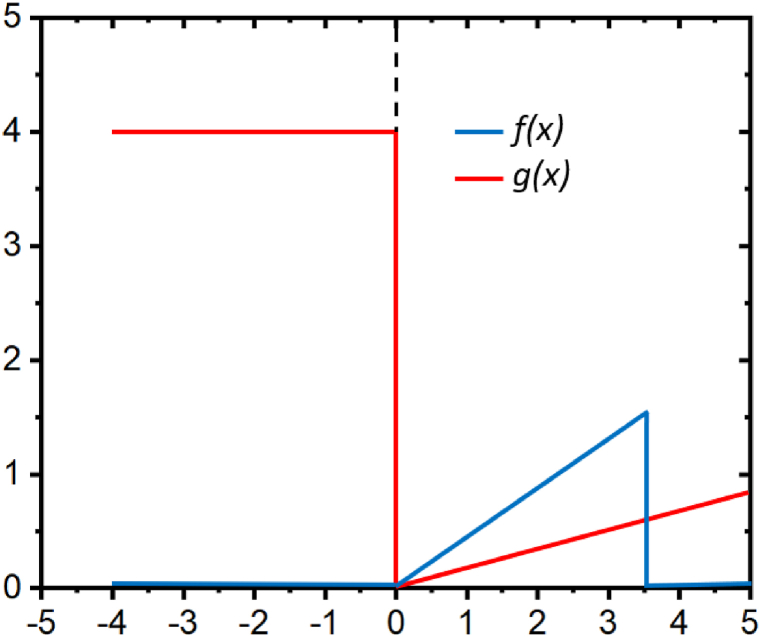
The functions of attachment and detachment, f and g, as delineated in the Huxley model.

It can be seen from Fig. 2 for *n*_*1*_, (*f(x)* = *0* since *x* < *0* and *g(x)* = *g*_*2*_ since *x* ≤ *0*). Substituting in equation (61), (62), (63), (64) yields

(62) $$-v\frac{dn_{1}}{dx}=-g_{2}n_{1}$$

(63) $$\frac{dn_{1}}{dx}=\frac{-g_{2}}{-v}n_{1}$$

(64) $$\frac{dn_{1}}{dx}-\frac{g_{2}}{v}n_{1}=0$$

Parameters used: *f*_*1*_ = 1:75, *g*_*1*_ = 0:58, *g*_*2*_ = 4 and *h* = 3.4.

We can solve this differential equation using the technique of an integrating factor (eq. (65)),

(65) $$n_{1}=Ae^{g_{2}\frac{x}{u}}\text{,}$$

for some constant *A* yet to be determined. Note that this solution is bounded as.

X → −∞ as it should be. Next, for $n_{2}(f\left(x\right)=f_{1}\frac{x}{h}$ since *0* < *x < h* and $g=g_{1}\frac{x}{h}$ since

X > *0)* substitute in eq. (61) to get (eq. (66), (67), (68)));

(66) $$-v\frac{dn_{2}}{dx}=\left(1-n_{2}\right)\frac{f_{1}x}{h}-n_{2}\frac{g_{1}x}{h}$$

(67) $$-v\frac{dn_{2}}{dx}+n_{2}\left(\frac{\left(f_{1}+g_{1}\right)x}{h}\right)=\frac{f_{1}x}{h}$$

(68) $$\frac{dn_{2}}{dx}-\frac{n_{1}}{v}\left(\frac{f_{1}+g_{1}}{h}x\right)=-\frac{f_{1}}{vh}$$

We solve it using the integrating factor (eq. (69));

(69) $$u=e^{-\int {\left(f_{1}+g_{1}\right)}^{\frac{x}{vh}}dx}=e^{-\left(f_{1}+g_{1}\right)\frac{x^{2}}{2hv}}=I\text{.}$$

Then multiplying the differential equation by I we get (eq. (70));

(70) $$\frac{d}{dx}(n_{2}e^{{-(f}_{1}+g_{1})\frac{x^{2}}{2hv}}=-\frac{f_{1}}{vh}\int xe^{{-(f}_{1}+g_{1})\frac{x^{2}}{2hv}}$$

Integrating both sides, we obtain (eq. (71), (72), (73), (74), (75), (76), (77), (78)));

(71) $$n_{2}\left(x\right)e^{-\left(f_{1}+g_{1}\right)}\frac{x^{2}}{2hv}=\frac{-f_{1}}{vh}\int xe^{-\left(f_{1}+g_{1}\right)\frac{x^{2}}{2hv}dx}$$

Let,

(72) $$u=e^{-\left(f_{1}+g_{1}\right)\frac{x^{2}}{2hv}}$$

(73) $$du=\frac{-\left(f_{1}+g_{1}\right)x}{hv}dx\text{,}$$

(74) $$xdx=\frac{hvdu}{-\left(f_{1}+g_{1}\right)}\text{,}$$

(75) $$\int e^{u}\frac{hvdu}{-\left(f_{1}+g_{1}\right)}=\frac{hv}{-\left(f_{1}+g_{1}\right)}\int e^{u}du=e^{u}+C_{2}\text{,}$$

(76) $$\frac{-f_{1}}{vh}\int xe^{-\left(f_{1}+g_{1}\right)\frac{x^{2}}{2hv}}dx=\frac{-f_{1}}{vh}\frac{hv}{-\left(f_{1}+g_{1}\right)}e^{-\left(f_{1}+g_{1}\right)\frac{x^{2}}{2hv}}+C_{2}$$

(77) $$\frac{-f_{1}}{vh}\int xe^{-\left(f_{1}+g_{1}\right)\frac{x^{2}}{2hv}}dx=\frac{f_{1}}{f_{1}+g_{1}}e^{-\left(f_{1}+g_{1}\right)\frac{x^{2}}{2hv}}+C_{2}$$

(78) $$n_{2}\left(x\right)=\frac{f_{1}}{f_{1}+g_{1}}+C_{2}e^{\frac{x^{2}\left(f_{1}+g_{1}\right)}{2hv}}$$

where C_2_ is a constant of integration. Finally, for $n_{3}(f\left(x\right)=0$ since *x > h* and $g\left(x\right)=g_{1}\frac{x}{h}$ since *x > h)* by inserting in (3.2.25) we have (eq. (79), (80)));

(79) $$-v\frac{dn_{3}}{dx}=n_{3}\frac{g_{1}x}{h}$$

(80) $$\frac{dn_{3}}{dx}+\frac{g_{1}x}{hv}n_{3}=0$$

We solve it using the integrating factor (eq. (81));

(81) $$n_{3}\left(x\right)=Ce^{\int \frac{g_{1}x}{hv}dx}=C_{3}e^{-\frac{g_{1}x^{2}}{2hv}}$$

**Fig. 3:**
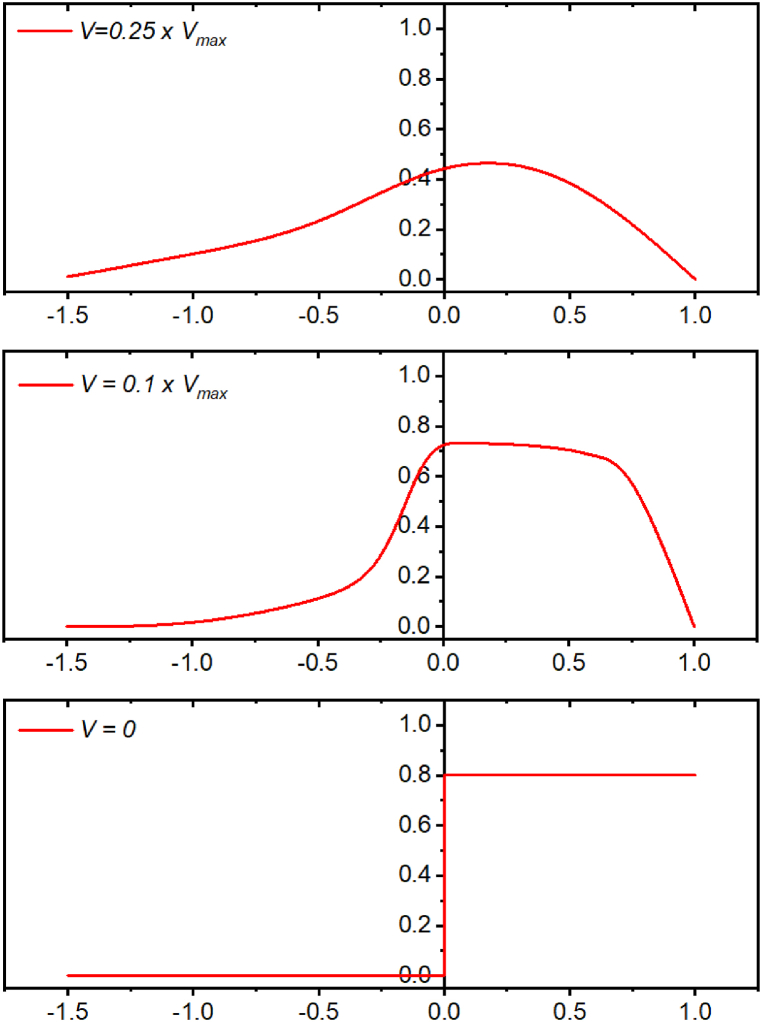
Steady state distribution of n for different values of v in the Huxley model.

For this to be bounded, the only solution is to be identically zero. In order to determine *c*_*1*_ and *c*_*2*_ we require that the solution be continuous at *x* = *0* and *x* = *h*, and thus *n*_*1*_*(0)* = *n2(0), n2(h)* = *0*. It follows that (eq. (82), (83), (84), (85)));

(82) $$n_{1}\left(0\right)=C_{1}=n_{2}\left(0\right)=\frac{f_{1}}{f_{1}+g_{1}}+C_{2};\;n_{2}\left(h\right)=0=C_{1}=\frac{f_{1}}{f_{1}+g_{1}}+C_{2}e^{\frac{h\left(f_{1}+g_{1}\right)}{2v}}$$

(83) $$C_{2}=-\frac{f_{1}}{f_{1}+g_{1}}e^{-\frac{\varphi }{V}}$$

where *φ* = *(f*_*1*_ + *g*_*1*_*)h/2*. Then

(84) $$C_{1}=\frac{f_{1}}{f_{1}+g_{1}}+C_{2}=\frac{f_{1}}{f_{1}+g_{1}}\left(1-e^{-\varphi /v}\right)$$

(85) $$\begin{array}{c} n\left(x\right)=\left\{ \begin{array}{ll} F_{1}\left(1-e^{-\varphi /v}\right)e^{\frac{x}{2h}G_{2}\frac{\varphi }{v}}, & x<0,\\ F_{1}\left(1-\frac{\varphi }{v}e^{\frac{x^{2}}{h^{2}}-1}\right), & 0<x<h,\\ 0, & x>h, \end{array}\right. \end{array}$$

where $F_{1}=\frac{f_{1}}{f_{1}+g_{1}}$ and $G_{2}=\frac{g_{2}}{f_{1}+g_{1}}$ are dimensionless. This steady solution is plotted in Fig. 3.

Plotted as a function of a dimensionless space. Parameters used: F_1_ = 0.81, G_2_ = 3.9,

φ = 400 and h = 10.

Finally, the force generated by the muscle can be calculated as a function of the velocity of contraction (eq. (86));

(86) $$p=\rho \int_{-\infty }^{\infty }kxndx=\rho \left(\int_{-\infty }^{0}\;kxndx+\int_{0}^{h}\;kxndx+\int_{h}^{\infty }\;kxndx\right)\text{.}$$

Solving this, we get the force-velocity relationship (eq. (87), (88), (89))),

(87) $$p=\frac{\rho kf_{1}h^{2}}{2\left(f_{1}+g_{1}\right)}\left(1-v/\varphi \left(1-e^{-\varphi /v}\right)\left(1+\frac{v}{2G_{2}^{2}\varphi }\right)\right)$$

where

(88) $$\varphi =\left(f_{1}+g_{1}\right)h/2\text{,}$$

has units of velocity and

(89) $$G_{2}=g_{2}/\;\left(h+g_{1}\right)$$

is dimensionless.

In general, the Huxley model is purely based on the kinetics of the cross-bridges and is the basis for many models of cardiac muscle behavior. Therefore, the accuracy of the Huxley model is limited by various assumptions, made by Huxley during its development. However, the Huxley model produced a more accurate analysis of the active contraction of the cardiac muscles, compared to the Hill model.

## 4 The Land model on human myocyte

The Land model involves the representation of cardiac muscle contraction, achieved through the analysis of tension development within human heart muscle cardiomyocytes; it was developed by Land et al. [17] using experimental data. The Land model integrates crucial components including tropomyosin kinetics, troponin C kinetics, the viscoelastic response of cells, and a three-state cross-bridge model that accurately captures the deformation of cross-bridges. Its calibration was accomplished using diverse experimental data acquired from human cardiomyocytes under physiological body temperature conditions. This data encompassed various aspects, such as the passive and viscoelastic traits of isolated myocytes, the relationship between steady-state force and calcium at different sarcomere lengths, and responses to rapid alterations in length, constant velocity shortening, and dynamic tension changes during length fluctuations in isolated myocytes. However, a notable dearth of experimental data from human cardiac myocytes at physiological body temperature posed challenges in quantitatively comprehending clinically relevant cardiac function and constructing comprehensive computational models of the entire organ. Specifically, essential measurements needed to characterize tension development variations in human cardiomyocytes occurring alongside alterations in cell length were unavailable. To address this gap, the researchers introduced an experimental dataset derived from skinned human cardiac myocytes. This dataset, employed within the Land model, stands as a pivotal characterization of the dependency of tension generation on length and velocity in human skinned cardiac myocytes under physiological body temperature conditions.

In this work, we will use the Land model to study the human myocyte contraction in detail, and in particular how different parameters affect myocyte contraction behaviors. First, we introduce the passive viscoelastic model, then the active tension model which includes thin Filament kinetics, the cross-bridges model and the active tension model. We will then examine the Land model and the relevant parameters that Land et al. used to establish the behavior of the cardiac muscle.

### 4.1 Passive viscoelastic model

The passive viscoelastic model for human cardiac myocytes was conceived through a meticulous analysis of the distinctive viscous behavior exhibited by these myocytes. This distinct viscosity pattern is attributed to the presence of titin molecules within the cardiac muscle's contractile machinery. It's noteworthy that this passive response is assumed to operate independently from other experimental data on active tension that this model encompasses.

In Land's conducted experiments [17], it was observed that the viscous component accounted for approximately 44 ± 5% of the total passive force. Of this force, a substantial 75% decayed within a span of 92 ± 24 ms. Notably, during shortening, the viscous response is notably diminished, possibly due to a considerably swifter decay rate and recovery occurring within the shortening interval. This passive behavior was effectively modeled using a three-parameter approach akin to a standard linear solid structure. This configuration involves a dashpot and a spring arranged in series, complemented by another spring situated in parallel. This choice of model was motivated by its simplicity and its capacity to capture both the passive and viscoelastic attributes of the response. In order to accurately replicate the well-established exponential force-length relationship commonly observed in prior investigations on isolated cells and cardiac tissue, a parallel spring mechanism was also incorporated (eq. (90), (91))).

(90) $$F_{1}=a\left(e^{bc}-1\right)$$

and has an exponential stress-strain relationship and:

(91) $$F_{2}=akC_{s}$$

is a linear spring.

The active contractile element T_A_ is introduced in later sections and has zero stiffness when the myocyte is not in an active contraction state. The dashpot element *F*_*d*_ has separate parameters for shortening and lengthening to accommodate the difference in viscous forces observed (eq. (92)).

(92) $$F_{d}=\left\{ \begin{array}{ll} a\eta_{l}\frac{dC_{d}}{dt}, & \frac{dC_{d}}{dt}>0,\\ a\eta_{s}\frac{dC_{d}}{dt}, & \frac{dC_{d}}{dt}<0 \end{array}\right.$$

This can be understood in analogy to electrical circuitry. Two elements in series have the same forces, which are *F*_*2*_ = *F*_*d*_ in our case (passive viscoelastic model), and the total strain (C) is the sum of two strains which are (eq. (93)):

(93) $$C=C_{s}+C_{d}$$

where *C*_*d*_ is the strain of dashpot and *C*_*s*_ is the strain of spring. This yields (eq. (94));

(94) $$\begin{array}{rr} F_{\text{total}}\left(c\right) & =F_{1}+F_{2}\text{,}\\ & =a\left(e^{bc}-1\right)+aKC_{s}\text{.} \end{array}$$

This represents the total force of the viscoelastic element (titin) as a function of the strain (C) (or extension ratio, λ = SL/SL_0_.) C and λ are related through (eq. (95));

(95) $$C=\lambda -1=\frac{SL}{SL_{0}}-1\text{.}$$

Furthermore, as *F*_*2*_ = *F*_*d*_ yields (eq. (96), (97)));

(96) $$F_{\text{total}}\left(c\right)=F_{1}+F_{d}\text{,}$$

(97) $$=a\left(e^{bc}-1\right)+\left\{ \begin{array}{ll} a\eta_{l}\frac{dC_{d}}{dt}, & \frac{dC_{d}}{dt}>0,\\ a\eta_{s}\frac{dC_{d}}{dt}, & \frac{dC_{d}}{dt}<0 \end{array}\right.$$

where s is for shortening and l for lengthening.

### 4.2 The active tension model

The cell's force-producing mechanism primarily comprises two fundamental filament types: thick filaments and thin filaments. Within the thin filaments, the constituents include actin, tropomyosin, and various troponins such as troponin C and troponin I. Meanwhile, the thick filaments are constructed from myosin cross-bridges. The generation of active tension stems from the interaction of myosin cross-bridges with binding sites located on actin within the thin filaments. These cross-bridges undergo a power stroke, characterized by the rotation of their head region, while concurrently inducing distortion in their tail region (the neck portion of the myosin molecule). The force generation is driven by the restoring forces exerted on this tail, which possesses spring-like characteristics. As the filaments undergo relative sliding, the reduction in distortion within the cross-bridge tail – induced by the power stroke – leads to a corresponding decrease in force output. To maintain optimal force generation, cross-bridges go through a cyclical process involving detachment, restoration to their original length, and subsequent attachment to fresh binding sites. This sequence facilitates the execution of another power stroke, thereby restoring their maximum force-producing capability.

#### 4.2.1 Thin filament kinetics

**Fig. 4:**
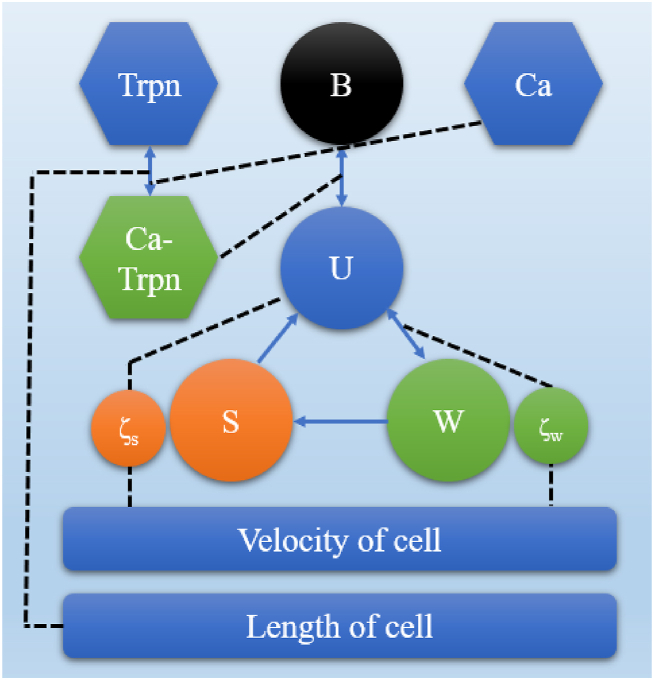
Cross-bridge dynamics during active contraction.

Fig. 4 describes the cross-bridge dynamics during active contraction, in which B represents the blocked state, which means Troponin and Tropomyosin cover the active site on actin (i.e., myosin head cannot bind to the active site on actin) [17]. During the resting phase, tropomyosin, which assumes a helical arrangement around the thin filament's actin, effectively obstructs the binding sites intended for myosin. This obstruction occurs due to the positioning maintained by troponin I, an additional protein situated on the thin filament. Upon cellular activation through electrical signals, a surge in calcium concentration transpires. Notably, calcium ions (Ca2+) from the cellular fluid adhere to the regulatory binding site found on troponin C. This calcium binding event triggers a structural shift in troponin C, resulting in the exposure of a binding pocket that troponin I can occupy. Consequently, troponin I relocates preferentially, disengaging itself from its role in retaining tropomyosin within the obstructive arrangement known as the ‘blocked’ state (designated as state U). In this state, the active sites situated on the actin become uncovered, thereby permitting the engagement of cross-bridges (that is, the attachment of myosin heads to the active sites on actin). This interaction forms cross-bridges, designated as state W. The W is the pre-power-stroke state which means the myosin head moves at the hinge region (Powerstroke state) causing the actin to slide past the myosin. ATP binds to the myosin head causing the myosin head to release the actin, with everything going back to the initial state (post-power-stroke stage, S) and generating tension. Furthermore, ζ_w_ and ζ_s_ are the mean distortions of the cross-bridge in the weak and strong states respectively.

The kinetics governing filaments are characterized by the interplay among different components, including calcium ions, troponin C, troponin I, and tropomyosin. These elements collaborate to regulate the accessibility of myosin-binding sites situated on actin. The activation process of the thin filaments exhibits notably cooperative tendencies, wherein even slight adjustments in intracellular calcium concentrations can potentially yield substantial variations in force generation. The primary biophysical explanation behind this cooperative behavior is connected to the end-to-end overlap of tropomyosin molecules. In view of optimizing applicability within multiscale modeling contexts, a straightforward and phenomenological depiction of cooperative activation is selected. This choice aligns better with the complexities of the multi-dimensional modeling tasks at hand. The dynamics of calcium ions binding is (eq. (98));

(98) $$\frac{dCaTRPN}{dt}=k_{TRPN}{\left(\frac{\left[Ca^{+2}\right]i}{{\left[Ca^{+2}\right]}_{T50}}\right)}^{nTRPN}\left(1-CaTRPN\right)-CaTRPN\text{,}$$

in which [Ca^2+^]_*i*_ is the intracellular calcium concentration. [Ca^2+^]_T50_ is the concentration of calcium required to bind 50% of troponin. Equation (98) can be solved analytically, with the following method: For simplicity, we write CaT instead of CaTRPN. We assume [Ca^2+^]_*i*_ is a constant and independent of time, then we can state (eq. (99), (100), (101), (102), (103), (104), (105), (106)));

(99) $${\left(\frac{\left[Ca^{+2}\right]i}{{\left[Ca^{+2}\right]}_{T50}}\right)}^{2}=a$$

So,

(100) $$\frac{\text{dCaT}}{0.1\left(a\left(1-\text{CaT}\right)-\text{CaT}\right)}=\text{dt}$$

(101) $$\frac{\text{dCaT}}{\left(a-\text{aCaT}-\text{CaT}\right)}=0.1\text{dt,}$$

(102) $$\frac{\log\left(a-\left(1+a\right)CaT\right)}{-\left(1+a\right)}=0.1t+C$$

(103) log⁡(a−(1+a)CaT)=−(1+a)0.1t+C

(104) $$a-\left(1+a\right)CaT=e^{\left(-\left(1+a\right)0.1t+C\right)}$$

with N being a constant.

(105) $$\text{CaT}\left(t\right)=\frac{a-{\text{Ne}}^{-\left(1+a\right)0.1t}}{1+a}$$

Using the initial condition t = 0 then:

(106) $$N=a-\left(1+a\right)CaT_{0}$$

**Fig. 5:**
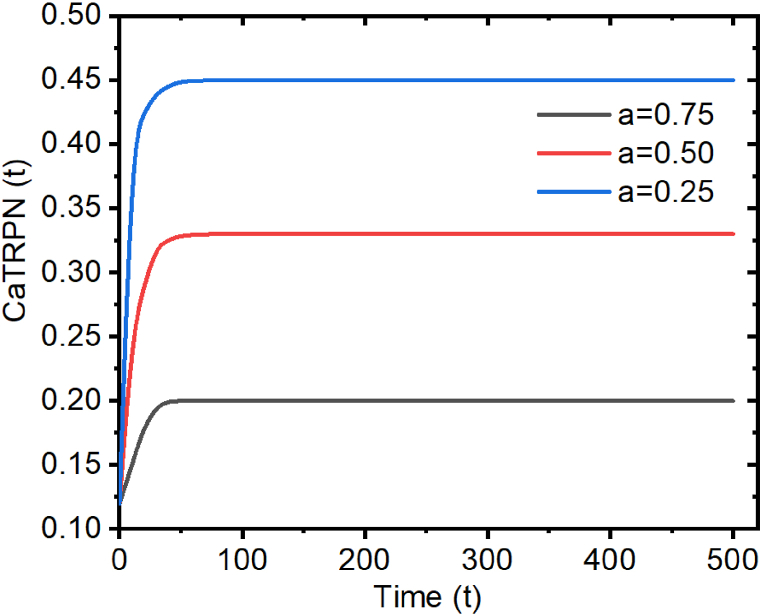
Diagram of CaTRPN for an arbitrary choice of parameters, when 0 < a <1.

• If [Ca^2+^]_*i*_ ≤ [Ca^2+^]_*T50*_ ⇒ 0 ≤ a ≤1, where (eq. (107));

(107) $$a={\left(\frac{{\left[{\text{Ca}}^{2+}\right]}_{i}}{{\left[{\text{Ca}}^{2+}\right]}_{\text{Ts}0}}\right)}^{2}$$

since 0 < C_a_T_0_ < 1 and 0 < a <1, then −1 < N < a <1, however 0 < N = e^c^ by construction so 0 < N < 1. The time course of CaTRPN for this case is shown in Fig. 5.

(It can be shown from Fig. 5, the CaTRPN(t) decreases when 0 < a <1 i. e the CaTRPN decreases when the intracellular calcium concentration ([Ca^2+^]_*i*_) is lower than the concentration of calcium required to bind 50% of troponin [Ca^2+^]T50).

**Fig. 6:**
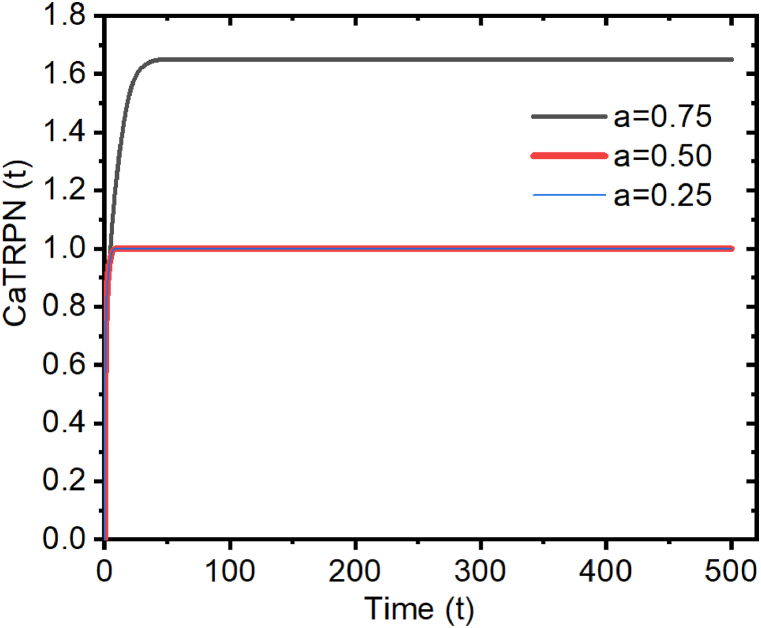
Diagram of CaTRPN for an arbitrary choice of parameters, when a >1.

• If [Ca^2+^]i > [Ca^2+^]T50 then a >1 and still 0 < N < a, so the corresponding time course of CaTRPN is shown in Fig. 6.

(It can be shown from Fig. 6, i.e., the CaTRPN initially increases then remains a constant to the value of 1, when the intracellular calcium concentration ([Ca^2+^]i) is higher than the concentration of calcium required to bind 50% of troponin ([Ca^2+^]T50).

CaTRPN signifies the proportion of troponin C units associated with calcium binding at its regulatory binding site. The parameter kTRPN, quantified at 0.1/ms, characterizes the pace of detachment. Meanwhile, nTRPN, set at 2, embodies the level of cooperativity in the rate of binding between calcium and troponin C. The parameter [Ca^2+^]_T 50_, which designates the activation midpoint, isn't uniformly consistent across different species. The troponin concentration CaTRPN serves as the driving force behind the alleviation of tropomyosin-induced blockage. This is expressed through the fraction of myosin-binding sites on actin that are obstructed, represented as B (eq. (108)):

(108) $$\frac{dB}{dt}=k_{b}{\text{CaTRPN}}^{\frac{nT_{m}}{2}}\cdot U-K_{u}\cdot {\text{CaTRPN}}^{\frac{nTm}{2}}\cdot B$$

Where kb and ku represent the transition rates for B and U states respectively. Equation (108) is not simple to solve analytically because it depends on the two variables U and B.

The states depicted in Fig. 4 encompass several distinct configurations, including the blocked state denoted as B, the state of unattached cross-bridges marked as U, the pre-power stroke state designated as W, and the force-generating state labeled as S. Notably, both states ‘W' and ‘S' within the framework contain an additional state designed to monitor the average distortion of cross-bridges when in these states. The progression between states ‘B' and ‘U' is instigated by the binding of calcium to troponin C. This interaction plays a pivotal role in driving the transition between these two states.

#### 4.2.2 The cross-bridges model

The force generation mechanism within the cross-bridges was formulated through the establishment of a three-state cycle. This cycle involves the cross-bridges transitioning through the states of being unbound (U) (eq. (109)), pre-power stroke (W), and post-power stroke (S). Additionally, the model incorporates a depiction of distortion decay, which tracks the mean distortion of cross-bridges when they are in the pre-power stroke (W) and post-power stroke (S) states. This comprehensive scheme is visually represented in Fig. 4.

(109) U=(1−B)−S−W

Equation (109) is obtained from the conversion of different states (eq. (110), (111))) shown in Fig. 4.

(110) $$\begin{array}{rr} & \frac{dW}{dt}=k_{uw}U-k_{wu}W-k_{ws}W-\gamma_{wu}W \end{array}$$

(111) $$\frac{dS}{dt}=k_{ws}W-k_{su}S-\gamma_{su}S$$

The parameters γ_su_ and γ_wu_ represent the cross-bridges unbinding rates in the pre-power stroke (W) and post-power stroke (S) states provided by a model of distortion decay. The distortion decay model is given by (eq. (112)):

(112) $$\gamma_{su}=\left\{ \begin{array}{ll} \gamma_{s}\left(-\eta_{s}-1\right), & \eta_{s}+1<0,\\ \gamma_{s}\eta_{s}, & \eta_{s}+1>1,\\ 0, & \text{if}\;\eta_{s}+1\epsilon \left[0,1\right] \end{array}\right.$$

Within this context, the parameters A_w_ and A_s_ correspond to the immediate reaction magnitude in response to distortion. Additionally, the parameters c_w_ and cs reflect the pace of distortion decay. It's important to note that variables marked with the subscript ‘w' are linked to the pre-power stroke state, denoted as W, while those with the subscript ‘s' pertain to the post-power stroke state, designated as S. Moreover, the variable λ signifies the cell length in relation to its resting length, as defined in Land's model. It is further assumed that the cross-bridges bind with no distortion in the pre-powerstroke state. In addition, the cross-bridges do not maintain the distortion when transitioning from W to S states with η_s_ = 0, indicating the absence of additional distortion to that particular power stroke.

In situations where alterations in cell length are absent, *η*_*w*_ = *η*_*s*_ = *0*. The total tension during active contraction is given by:

T_a_ = number of cross-bridges ∗ cross-bridges stiffness ∗ powerstroke ∗ distortion ∗ S. When distortion in both powerstroke is considered, the total tension is given by (eq. (113)):

(113) $$T_{a}=\left(\zeta_{s}+1\right)S+\eta_{w}W$$

The component accounting for distortion-induced detachment has already been integrated into equations (111), (112)), corresponding to the rates of change for dW/dt and dS/dt, respectively. In reaction cycles like the one adopted in our cross-bridges model, it's often more convenient to establish parameterization based on the equilibrium occupation of states and an encompassing cycling rate, which regulates both forward and reverse rates. The equilibrium occupation can be more readily defined with the aid of a prior or reference value, as it exhibits a lesser degree of dependence on the species. In contrast, the overall cycling rate governs the kinetics of tension development and is prone to significant variation across species. Consequently, the subsequent definitions are introduced.

• *r*_*s*_ - steady-state duty ratio S/(U + W + S)
• *r*_*w*_ - the equilibrium proportion between the pre-powerstroke and non-strongly bound states = steady-state W/(U + W)

TRPN50 = value of CaTRPN, where B = 0.5 in steady state.

The provided explanations and numerical values facilitate the model's parameterization due to the convenience of establishing accurate initial approximations for *r*_*s*_, *r*_*w*_, and TRPN50. By presuming *r*_*s*_ = 0.25, TRPN50 = 0.35, and *r*_*w*_ = 0.5, the resultant parameters, pivotal for the model equations, are deduced as follows (eq. (114), (115), (116))):

(114) $$k_{wu}=k_{uw}\left(1/r_{w}-1\right)-k_{ws}$$

(115) $$k_{su}=k_{ws}r_{w}\left(1/r_{s}-1\right)$$

(116) $$k_{b}=k_{u}{\text{CaTRPN}}^{nTm}/\left(1-r_{s}-\left(1-r_{s}\right)r_{w}\right)$$

in equation (112), the magnitude of immediate distortion within the W and S states is considered equal, as these states correspond to the distortion resulting from the relative motion of the filaments (eq. (117)),

(117) $$A_{s}=A_{w}=A_{eff}r_{s}/\left(\left(1-r_{s}\right)r_{w}+r_{s}\right)$$

Assuming the distortion decay rates to be proportional to steady-state cross-bridges cy-cling rates (eq. (118), (119))):

(118) $$c_{w}=\varphi k_{uw}U/W=\varphi k_{uw}\left(\left(1-r_{s}\right)\left(1-r_{w}\right)\right)/\left(\left(1-r_{s}\right)r_{w}\right)$$

(119) $$\begin{array}{rr} c_{s} & =\varphi k_{ws}W/S=\varphi k_{ws}\left(\left(1-r_{s}\right)r_{w}/r_{s}\right) \end{array}$$

Four states include Blocked, Unbound, Weak and Strong. These are all possible states of the cross-bridge. The weak and strong states differ from the other states in that have a mean distortion associated with them, ζ_w_ and ζ_s_. Therefore, this model explains the changes of the four states, as well as the changes of the mean distortion.

### 4.3 The length-dependence of tension

Another pivotal aspect concerning tension development in cardiac physiology pertains to the augmentation in force generation when myocyte length experiences an increment. This phenomenon operates at the organ level, manifesting as the renowned ‘Frank-Starling’ effect [2,15,17,21,48,48]. This effect signifies that an elevation in end-diastolic volume induces an increase in the volume of blood expelled during ejection, thus maintaining a harmonious inflow-outflow equilibrium within the heart. Nevertheless, the comprehensive elucidation of the molecular mechanisms governing these phenomena remains incomplete. In this context, Land et al. [13] postulated that the phenomenological model encapsulated the cellular repercussions, characterized by a shift in intracellular calcium sensitivity and an enhancement in the maximum tension attainable in response to elongation, as illustrated below (eq. (120), (121), (122))):

(120) $${\left[{\text{Ca}}^{2+}\right]}_{T50}={\left[{\text{Ca}}^{2+}\right]}_{T50}^{ref}+\beta_{1}\left(m\left(\lambda ,1.2\right)-1\right)$$

(121) $$h\left(\lambda \right)=m(0,h^{\prime }\left(m\left(\lambda ,1.2\right)\right)$$

(122) $$h^{\prime }\left(\lambda \right)=1+\beta_{0}\left(\lambda +m\left(\lambda ,0.87\right)-1.87\right)$$

within this framework, the parameter *β*_*0*_ embodies the alteration in maximum tension, influenced by shifts in filament overlap, while *β*_*1*_ encapsulates the modification in calcium sensitivity. Consequently, the aggregate active tension of the entire model is expressed as follows (eq. (123)):

(123) $$T_{a}=h\left(\lambda \right)\frac{T_{ref}}{r_{s}}\left(\left(\xi_{s}+1\right)S+\xi_{w}W\right)\text{,}$$

where T_ref_ is the maximal active tension at resting length.

Length dependency pertains to the intricate interplay among sarcomere length, calcium dynamics, and cross-bridge behavior throughout the contraction process. Within cardiomyocytes, as the sarcomere length extends during contraction, the engagement of cross-bridges becomes more pronounced. The fundamental explanations underlying this phenomenon are rooted in two theoretical possibilities: an augmentation in the count of engaged cross-bridges or an elevation in cross-bridge strain. Moreover, an increased rate of cross-bridge attachment results in a higher number of engaged cross-bridges. Simultaneously, a reduced rate of cross-bridge detachment or an elevated calcium concentration in the sarcoplasm contributes to the heightened engagement of cross-bridges. It's noteworthy that while the concentration of calcium significantly influences this process, it remains independent of the length of the sarcomeres [3].

Modifying the length of sarcomeres yields ramifications on the attachment and detachment rates of cross-bridges to the exposed and activated binding sites on actin. Given that a muscle fiber's volume remains constant through length variations, the fiber's diameter must increase when at a shorter length [4,50,53,54]. Consequently, the hexagonally arranged lattice of myofilaments within each sarcomere experiences a wider spacing, leading to a change in the gap between myosin heads and the thin filaments. This alteration influences their interaction in the formation of cross-bridges [18]. The myosin heads are positioned at the extremities of articulated arms encompassing myosin light chains, these arms are oriented away from the longitudinal axis of the thick filament. When the sarcomere lengthens, the myosin heads can conveniently fit between the thick and thin filaments, allowing for swift binding and the formation of cross-bridges. This proximity between myosin heads and actin-binding sites also augments the robustness of the actomyosin bond, thereby decreasing the likelihood of cross-bridge detachment. Conversely, at shorter lengths, the positioning of myosin heads becomes less favorable, and their binding might also be hindered by the overlapping configuration of the thin filaments [38]. These influences are particularly pronounced at lower levels of activation, where binding sites activated on the thin filaments are relatively scarce [17].

The activation of the cross-bridges is measured as a percentage of the maximum tension generation for a specific amount of overlap of the myofilament. The change in sarcomere length also influences the kinetics of calcium which control activation and contraction in the cardiac muscle. The cisterns responsible for releasing calcium demonstrate an apparent tethering to the Z-plates, positioned around the midpoint of the actin-myosin overlap, particularly when the muscle is at its optimal length. However, when the muscle lengthens, the dispersion of calcium tends to occur over extended distances to access the actin-binding sites [54]. Consequently, this scenario results in elongated rise periods during the calcium activation phase of the contraction [17]. Furthermore, the interplay with muscle length has a proportional effect on the rates of cross-bridge attachment and detachment. Specifically, an increase in sarcomere length leads to a corresponding elevation in the rate of cross-bridge attachment, while the rate of detachment diminishes proportionally.

## 5 Results

**Fig. 7:**
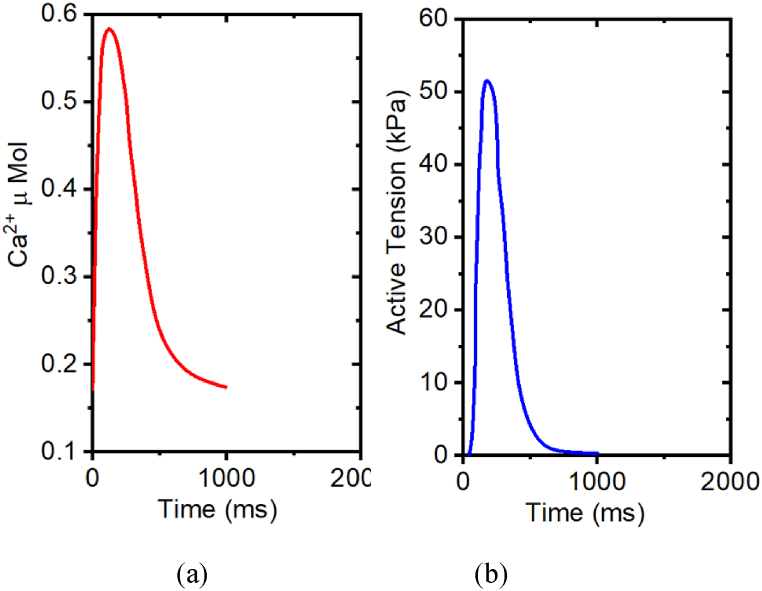
Isometric contraction with stretch ratio λ = 1.0, using prescribed intracellular calcium transients (a) Ca2+ plot, (b) active tension plot.

The Myocyte model is implemented in MATLAB and solved using an ode15 solver with adaptive time stepping. An ode-15 solver was chosen because the mathematical model is stiff. A problem is categorized as “stiff” when the sought-after solution exhibits gradual variations, yet neighboring solutions exhibit rapid fluctuations, necessitating the numerical method to employ small increments to achieve satisfactory outcomes. This model serves as an illustration of stiffness. The syntax for ode-15 is, [t,y] = ode15s(odefun, tspan, *y*_*0*_), where tspan = [t_0_t_f_ ]. The solver integrates the system of differential equations y = f(t,y) from t_0_ to t_f_ with initial conditions *y*_*0*_. Each row in the solution array y corresponds to a value returned in the column vector. We first examine the isometric contraction with stretch ratio λ = 1.0, using prescribed intracellular calcium transients shown in Fig. 7(a). Fig. 7(b) is the corresponding developed tension. Active tension increases with increased Ca^2+^, and reaches the peak tension (50 kPa) at 220 ms, while Ca^2+^ reaches peak at 120 ms, which is earlier than the peak tension. After reaching the peak, T_a_ decreases with decreased intracellular Ca^2+^.

**Fig. 8:**
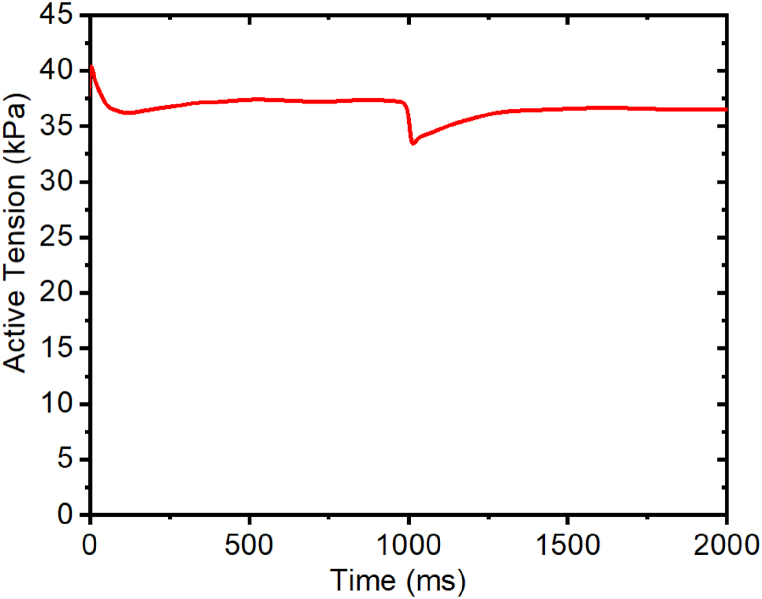
Effects of the calcium parameter on the tension in the cardio myocytes for an isotonic experiment.

Secondly, we model the release experiment, in which the intracellular Ca^2+^ is maintained with a constant level (30μMol), and the myocyte is stretched to 1.005 within 10 ms, then held with fixed length at a stretch ratio until 1000 ms, and then allowed to contract with a constant contraction velocity within 5 ms to return to its rest length, or λ = 1.0. Finally, the myocyte is held at a constant stretch ratio of 1.0 until 2000 ms. Fig. 8 shows the time course of active tension during all of the release experiments. From Fig. 8, we can see that the active tension increases quickly in the beginning because of the change in stretch ratio from 1 to 1.005 in 10 ms, and that later it decreases since the stretch is fixed at a constant value, gradually reaching a steady state with an active tension of 37 kPa. From 1000 ms to 1005 ms, the myocyte shortens to its rest length, because the active tension is velocity dependent. Thus, it decreases with a minimum value of 33 kPa. After 1005 ms, since the stretch ratio is fi again, the active tension recovers to a higher level and reaches a steady state after 1500 ms, with the final active tension being around 36.5 kPa.

**Fig. 9:**
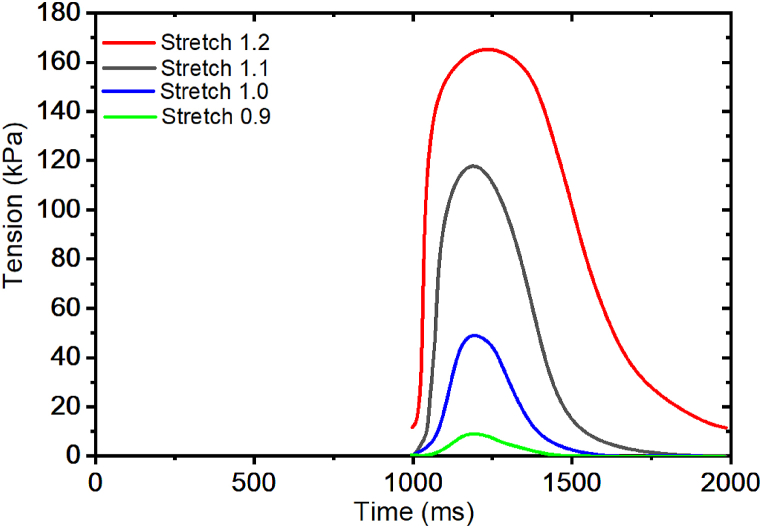
Isometric contraction of the cardiac muscle at different stretch ratios: 0.90, 1.00, 1.10 and 1.20.

We examine the isometric contraction of the cardiac muscle at different stretch ratios: 0.90, 1.00, 1.10, and 1.20, using the intracellular calcium transients described in Fig. 7(a–b). Fig. 9 shows the corresponding active tensions developed at the different stretch ratios. Active tension developed in the muscle increases steadily as Ca^2+^ transient increases up to the peak tensions of 10 kPa, 50 kPa, 120 kPa and 170 kPa at different stretch ratios: 0.90, 1.00, 1.10 and 1.20 respectively. However, the Ca^2+^ transient reaches a peak at 0.6 μM, which is higher than the amount required for achieving the highest peak tension at 1.2 stretch ratios of 0.55 μM Ca^2+^ transient.

**Fig. 10:**
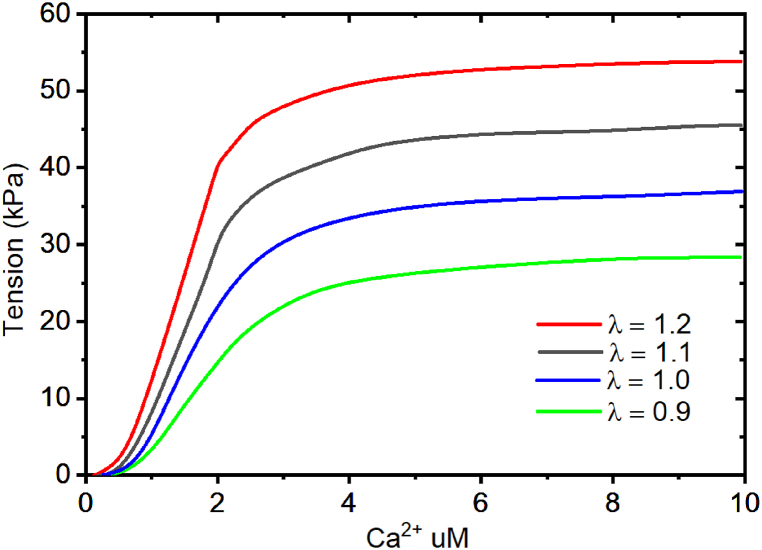
The relationship between the level of Ca^2+^ and the tension formed in the cardiac muscles during active contraction at different sarcomere lengths.

We then examine the isometric behavior of the tension developed in the muscle due to variation of the intracellular calcium profile at different stretch ratios. The intracellular Ca^2+^ varies with different values of the stretch. In this case, we were able to establish the effects of stretch ratios and Ca^2+^ on the tension developed in the muscle as shown in Fig. 10. The tension developed at the stretch ratio of 0.9 is 20 kPa, while the tension developed at 1.0 stretch ratio is 30 kPa. In addition, the tension developed at a stretch ratio of 1.1 and 1.2 is 40 kPa and 50 kPa respectively. It is observed in Fig. 10 that a higher stretch ratio contributes to the development of highly active tension in the muscle. Moreover, the active tension increases steadily, initially with increased intracellular calcium at different stretch ratios up to specific peak values.

## 6 Discussion

### 6.1 Parameter sensitivity study (β_0_ parameters)

Table 1 illustrate the data used for studying the sensitivity of different parameters in the myocyte model.

**Table 1:** Selected parameters for an Intact Myocyte.

TRPN 50	[Ca^2+^]_T50_	*β*_*0*_	*Β*_*1*_	Tref	Relative change
0.18	0.40	1.15	−1.2	60.2	−50
0.26	0.60	1.73	−1.8	90	−25
0.35	0.81	2.30	−2.4	120	0
0.44	1.01	2.88	−3.0	150	25
0.53	1.21	3.45	−3.6	180	50
0.70	1.61	4.60	−4.8	240	100

**Fig. 11:**
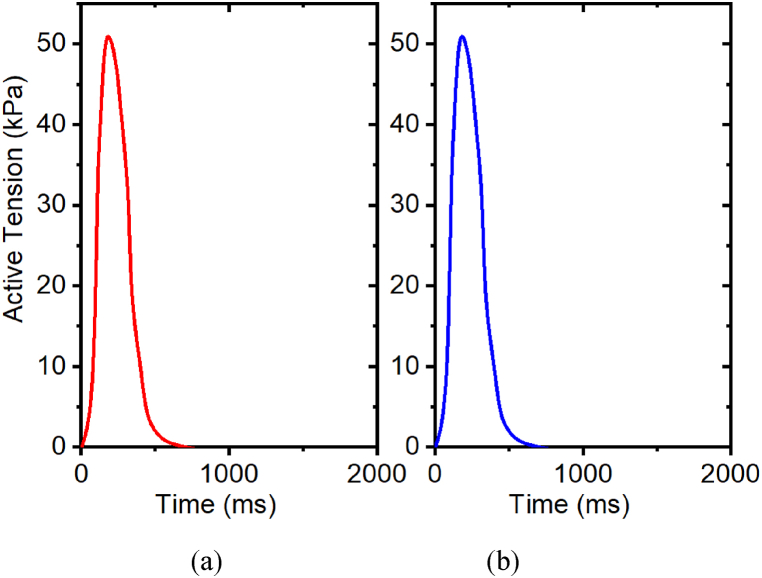
Effects of β0 on active tension development with stretch ratio 1.0, (a) β_0_ = 1.15; (b) β_0_ = 4.6. The prescribed calcium profiles are same for all simulations, as shown in Fig. 7(a–b).

*β*_*0*_, a parameter contingent on length, embodies the alteration in maximum tension, driven by modifications in filament overlap, reported in Table 2. The isometric active contraction with a fixed stretch ratio of 1.0 is studied in intact myocyte cells, regarding the variation of the *β*_*0*_ parameters and its respective effect on the tension developed in the muscle. For *β*_*0*_, in Fig. 11, the left side being active tension from *β*_*0*_ = min_value_, which is 1.15, and the right side being *β*_*0*_ = max_value_, which is 4.6. With increased *β*_*0*_, the peak active values of tension developed in the muscle remain constant. Based on these results, for intact myocyte cells, the value of the maximum tension attained remains constant at all levels of *β*_*0*_ parameter (i.e., it does not affect peak tension since in the simulation the length is fixed for isotonic tension experiments).

**Table 2:** Effects of *β*_*0*_ on peak active tension with stretch ratio 1.0.

*β*_*0*_	Intact Myocyte	
	Peak active tension (kPa)	Relative change
1.15	50	0%
1.73	50	0%
2.30	50	0%
2.88	50	0%
3.45	50	0%
4.60	50	0%

### 6.2 β_1_ parameters

**Fig. 12:**
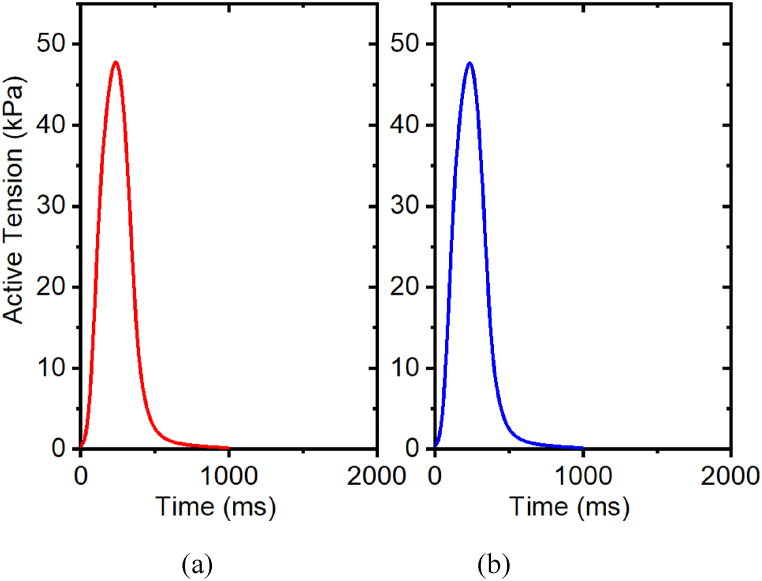
Effects of *β*_*1*_ on active tension development with a stretch ratio 1.0, (a) *β*_*1*_ = −1.2; (b) *β*_*1*_ = −4.8. The prescribed calcium profile is the same for all simulations, as shown in Fig. 7(a–b).

Table 3 shows the effect of *β*_*1*_ on peak active tension. The *β*_*1*_ is a length-dependent parameter, which captures the change in calcium sensitivity. The *β*_*1*_ is a length-dependent parameter, which captures the change in calcium sensitivity. The isometric active contraction, with a fixed stretch ratio 1.0, is studied in intact myocyte cells, with reference to the variation of the *β*_*1*_ parameters and its respective effect on the tension developed in the muscle. For *β*_*1*_, in Fig. 12(a–b), the left side being active tension from *β*_*1*_ = min_value_, which is −1.2, and the right side being *β*_*0*_ = max_value_, which is −4.8. With increased *β*_*1*_, the peak active values of tension developed in the muscle remain constant. Based on these results, for intact myocyte cells, the value of the maximum tension attained remains constant at all levels of the *β*_*1*_ parameter (i.e., it does not affect peak tension since in the simulation the length is fixed for isotonic tension experiments).

**Table 3:** Effects of *β*_*1*_ on peak active tension with stretch ratio 1.0

*β*_*1*_	Intact Myocyte	
	Peak active tension (kPa)	Relative change
−1.2	50	0%
−1.8	50	0%
−2.4	50	0%
−3.0	50	0%
−3.6	50	0%
−4.8	50	0%

### 6.3 [Ca^2+^]_T50_ parameter

**Fig. 13:**
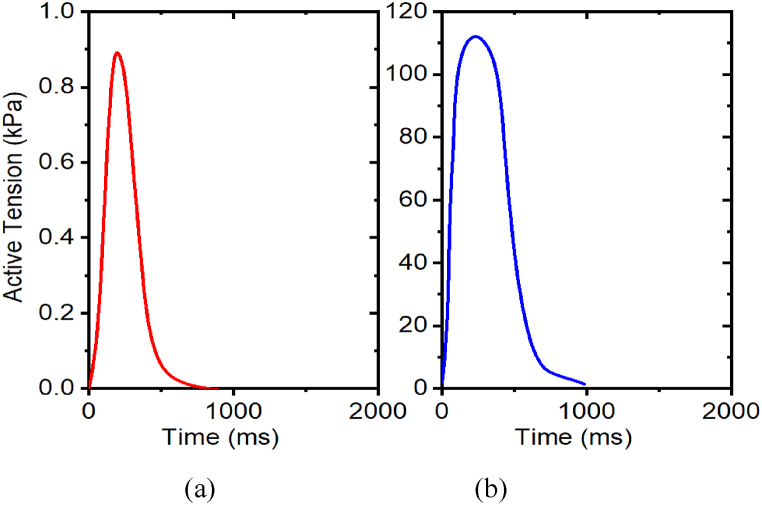
Effects of [Ca^2+^]_T50_ on active tension development with stretch ratio 1.0, (a) [Ca^2+^]_T50_ = 0.40; (b) [Ca^2+^]_T50_ = 1.61. The prescribed calcium profile is the same for all simulations, as shown in Fig. 7(a–b).

Further, we studied the isometric behavior of the [Ca^2+^]_T50_ and how it influences the amount of tension generated in cardiac muscles as shown in Table 4. The effects of [Ca^2+^]_T50_ on the tension developed in the muscle depend on the amount of stretch ratio 1.0. The tension developed in the muscle increased initially with a decrease of the [Ca^2+^]_T50_ parameter profile. The isometric effects of the [Ca^2+^]_T50_ on the tension developed in the cardiac muscle is presented in Fig. 13(a) and (b). On the right side is the active tension developed at [Ca^2+^]_T50_ = 0.40 on a stretch ratio of 1.0. The maximum tension achieved at this [Ca^2+^]_T50_ level is 115 kPa. The sub-figure on the right represents the tension developed in the intact myocyte at 1.61 μM of [Ca^2+^]_T50_. The maximum tension developed at this calcium [Ca^2+^]_T50_ is 0.9 kPa on a stretch ratio of 1.0. Based on these results, for intact myocyte cells, the value of the peak active tension decreases with increased [Ca^2+^]_T50_ parameter on a stretch ratio of 1.0.

**Table 4:** Effects of [Ca^2+^]_T50_ on peak active tension with stretch ratio 1.0

[Ca^2+^]_T50_	Intact Myocyte	
	Peak active tension (kPa)	Relative change
0.40	115	113%
0.60	95	90%
0.81	50	0%
1.01	16	−68%
1.21	4.4	−91.2%
1.61	0.9	−98.2%

### 6.4 T_ref_ parameter

**Fig. 14:**
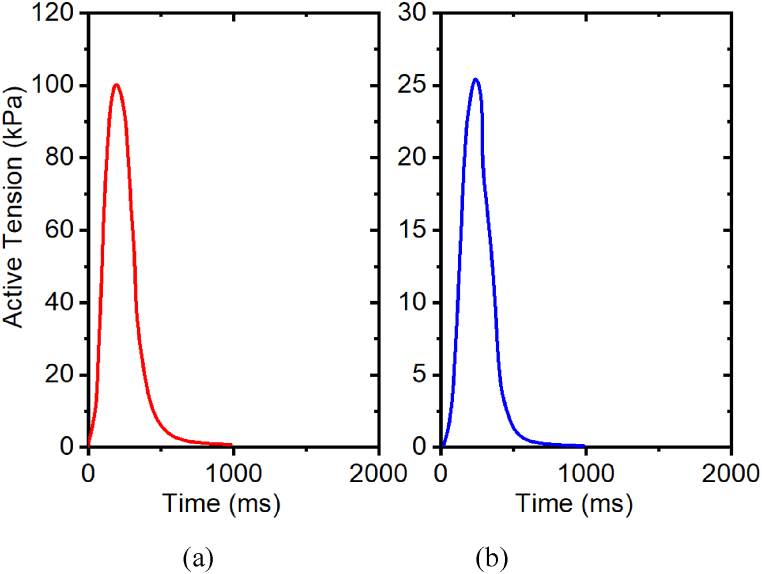
Effects of T_ref_ on active tension development with stretch ratio 1.0, (a) T_ref_ = 60.25; (b) T_ref_ = 240. The prescribed calcium profile is the same for all simulations, as shown in Fig. 7(a–b).

Table 5 shows the effect of T*ref* on peak active tension. We examined the isometric effects of the T*ref* parameter on the tension developed in the intact myocyte cells. Fig. 14(a–b) contains two subfigures that illustrate the effects of the T*ref* parameter on the tension developed in the muscle. We observe that the maximum active tension achieved is 60.25 kPa for the intact myocyte at T*ref* = 25, based on the sub-figure on the left side. On the other hand, the sub-figure on the right shows that the peak active tension is 100.5 kPa when the T*ref* is 240 kPa. The peak active tension increases with increased T*ref* parameter in the intact myocyte. Based on these results, the peak active tension increases with an increased T*ref* parameter in the intact myocyte with stretch ratio of 1.0.

**Table 5:** Effects of Tref on peak active tension with stretch ratio 1.0

Tref	Intact Myocyte	
	Peak active tension (kPa)	Relative change
60.25	25	−50%
90	39	−22%
120	50	0%
150	61	22%
180	79	58%
240	100.5	101%

### 6.5 TRPN50 parameter

**Fig. 15:**
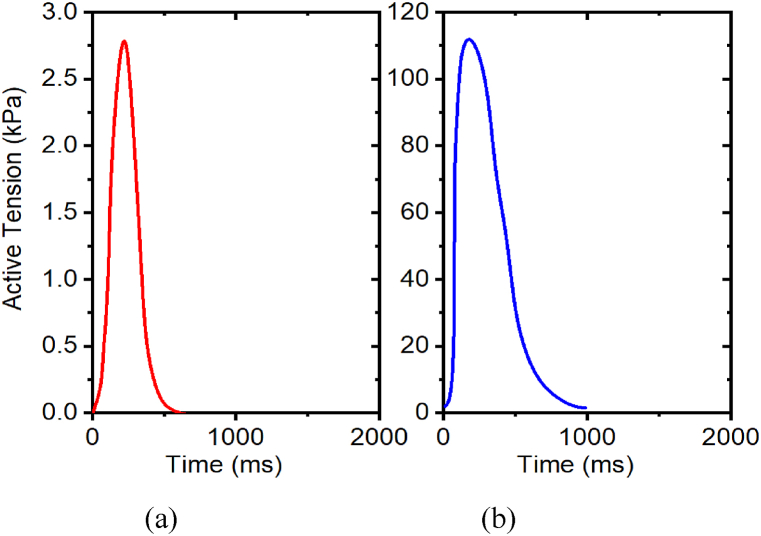
Effects of TRPN50 on active tension development with stretch ratio 1.0, (a) TRPN 50 = 0.18; (b) TRPN 50 = 0.7. The prescribed calcium profile are the same for all simulations, as shown in Fig. 7(a–b).

Table 6 illustrates the effect of TRPN50 on peak active tension. We studied the effects of the TRPN50 parameter on the tension developed in the cardiac muscle, examining the parametric behavior of the tension by varying the TRPN50 parameter at stretch ratio 1.0. According to sub- Fig. 15(a)–15(b), the tension developed in the muscle increased initially with a decrease in the TRPN50 parameter profile. Based on these results, for intact myocyte cells, the value of the peak active tension decreases with the increased TRPN50 parameter on a stretch ratio of 1.0.

**Table 6:** Effects of TRPN50 on peak active tension with stretch ratio 1.0

TRPN50	Intact Myocyte	
	Peak active tension (kPa)	Relative change
0.18	112	124%
0.26	90	80%
0.35	50	0%
0.44	24	−52%
0.53	10.2	−79.6%
0.70	2.8	94.4%

### 6.6 Functionality and limitations of the model

The Land model, harnessed in this investigation, adeptly reproduces the saturation of force (tension) for corresponding sarcomere lengths, exhibiting notable congruence with experimental observations. Nevertheless, discernible differences arise in terms of simulated versus experimental sensitivity to external Ca^2+^ concentrations. Notably, the simulated force tends to reach saturation at lower Ca^2+^ levels, a divergence attributed to the decreased Ca^2+^ sensitivity in skinned cardiac cells in comparison to their intact counterparts. Moreover, the present model effectively captures the shift in Ca^2+^ sensitivity evident in steady-state force-calcium relationships across diverse sarcomere lengths (as depicted in Fig. 10). This shift is manifest in normalized force-calcium relationships. Simulation outcomes affirm that an elongation of the sarcomere results in diminished half-saturation Ca^2+^ values, thereby indicating heightened Ca^2+^ sensitivity (as illustrated in Fig. 7(a–b). Importantly, this observed shift in Ca^2+^ sensitivity has also been corroborated experimentally in mouse cardiac cells.

However, it is essential to acknowledge certain limitations inherent in the cardiac myocyte contraction model due to the deliberate simplification of the biophysical contraction mechanism. Specifically, the model employs a simplified rendition of the relationship between contraction force and cellular shortening, following Hooke's law, whereas the actual dependence is more intricate. Furthermore, the model does not account for the impact of cellular shortening on Ca^2+^ transients, unlike the Land model. Nonetheless, this effect remains relatively minor. It's worth noting that like numerous other models, this one neglects intracellular spatial inhomogeneities in Ca^2+^ concentration and cross-bridges binding sites.

## 7 Conclusion

Crafting physiologically realistic heart models is a complex endeavor fraught with numerous challenges [5]. These challenges encompass intricate anatomical geometries, nonlinear material responses exhibited by the myocardium, fluid-structure interactions, and intricacies spanning different length and function scales. Over the past several decades, the finite element (FE) method has been employed to forge many nonlinear models of cardiac mechanics, addressing these multifaceted complexities. Comparable to nerve cells, muscle cells exhibit the ability to conduct action potentials along their membrane surfaces, while also translating these electrical signals into mechanical contractions, thus facilitating the execution of work. The scope of this endeavor was dedicated to an in-depth exploration of mathematical models governing the active contraction of cardiac myocytes. Diverse models have been devised, drawing inspiration from existing research and various conceptual frameworks underpinning the operation of human cardiac muscle [10]. The essential role played by cardiac muscles in enabling the heart's functionality lies in their capacity to contract and propel blood flow to distinct parts of the body. In this pursuit, the initial focus involved a comprehensive review of the Hill and Huxley models applicable to general muscle cells. The research subsequently concentrated on mathematical modeling specific to myocytes, employing the Land model as a cornerstone. Notably, the Land model derives its foundations from real-world experimentation. Mathematical simulations undertaken within this project were grounded in the constructs presented by the Land model. The Hill model was also scrutinized, its examination informed by the results of mathematical analyses pertaining to cardiac muscle contraction. This model establishes a relationship between force and velocity within cardiac myocytes [69]. However, the Hill model does exhibit limitations, chiefly stemming from its lack of incorporation of sarcomere element interactions during its development. Originating prior to the comprehensive understanding of sarcomere anatomy, the Hill model was founded upon Hill's observations indicating that the correlation between the constant rate of shortening velocity and load during muscle contraction against a consistent load can be effectively characterized by the force-velocity equation.

The intent of the Land model was to achieve a delicate equilibrium between modeling intricacy and the feasibility of parameterization through available data. Within our study, we conducted simulations involving various parameters as outlined in the Land model, endeavoring to elucidate the tension development dynamics within the cardiac myocytes of the human heart. The validation of the model hinged upon a substantial body of experimental data, which extensively explored the impact of diverse parameters—such as calcium, T_ref,_ and *β* —on the tension emerging within myocyte cells, all within the context of physiological room temperature. Numerous models dedicated to the contraction of cardiac myocytes have been formulated over time. However, prior iterations often omitted the incorporation of sarcomere shortening during a twitch. Instead, their emphasis predominantly centered on simplifying the depiction of cross-bridge kinetics, their interplay with Ca^2+^ dynamics, and meticulous replication of established experimental data concerning both steady-state and dynamic force-calcium relationships. Admittedly, the utilization of the Land model carries certain limitations. Firstly, it employs a streamlined representation of the connection between contraction force and cellular shortening, grounded in Hooke's law. This approach contrasts with the more intricate actual dependence that exists. Secondly, the model fails to account for the ramifications of cellular shortening on Ca^2+^ transients—a shortcoming paralleling that of the Land model—though the impact of this omission remains relatively negligible. Lastly, akin to a majority of models, this one disregards the spatial inhomogeneities prevalent in intracellular Ca^2+^ concentration and cross-bridge binding sites.

This investigation delved into the mechanics of myocyte contraction within the human cardiac muscle, leveraging the Land model as a foundational framework for the systematic analysis of parameters influencing the development of muscle tension. A comprehensive exploration of diverse facets within the human heart was undertaken, with a specific focus on the intricate composition of myocyte cells. This examination aimed to unravel the intricate interplay between various parameters intrinsic to the cardiac muscle's mechanics. Furthermore, a detailed exploration encompassed earlier models, including the Hill and Huxley models, which significantly contributed to shaping the evolution of the Land model. A rigorous review of the existing literature was conducted to glean insights into the mathematical modeling of myocyte cardiac cells. Noteworthy among these was the Hunter model, offering an encompassing comprehension of passive-active mechanics in cardiac muscle, with applications extending to continuum mechanics models of the heart. Additionally, scrutiny was directed towards the Rice Filament model, which approximated activation and force generation in cardiac myofilaments and delineated the intricate interlinkages between mechanical contraction and electrical excitation in cardiac cells. These foundational models, along with their mathematical formulations, formed the bedrock for comprehending the intricate processes underpinning excitation and contraction in cardiac cells. The focal point of this study rested on the Land model, seeking to simulate the dynamic contraction of real human myocytes. Through its application, successful simulation of myocyte contraction was realized across diverse conditions, encompassing both isometric and isotonic tensions. Notably, our findings unveiled a direct correlation between stretching and the attainment of peak active tension, aligning with the well-established paradigm of length-dependent tension generation.

Five parameters were selected: [Ca^2+^] T50, Tref, TRPN50, β0 and β1. Each parameter was varied between −50% and 100%, to examine the isometric effects of each parameter on the behavior of the tension developed in the intact myocyte cells, with the most sensitive parameter being [Ca^2+^]T50. In conclusion, it was found that the Land model provides a good platform for the analysis of the active contraction of the human cardiac myocyte.

## Data availability

The data used to support the findings of this study are included within the article.

## Funding

This work is supported by the Ministry of Research, Innovation and Digitization through Program 1—Development of the national research-development system, Subprogram 1.2—Institutional performance-projects for financing excellence in 10.13039/100006529RDI, contract no. 28PFE/December 30, 2021.

## Author contributions

Conceptualization, F.A., M.I.H.S., D.D, M.A.A. and D.R.D.; methodology, F.A., M.A.A., T.A. and D.R.D; software, F.A., D.R.D.; formal analysis, F.A., M.I.H.S, D.D, R.C. and D.R.D; investigation, F.A., M.A.A., D.D and D.R.D; resources, F.A., M.I.H.S., T.A. and R.C., D.D.; writing—original draft preparation, F.A., M.I.H.S., D.D and D.R.D; writing—review and editing, F.A., M.I.H.S., D.D, R.C. and D.R.D.; funding acquisition, D.D. All authors have read and agreed to the published version of the manuscript.

## Declaration of competing interest

The authors declare that they have no known competing financial interests or personal relationships that could have appeared to influence the work reported in this paper.
